# Preclinical Evaluation of Berry Extracts as a Nutritional Intervention to Alleviate Myocardial Ischemia–Reperfusion Injury and Atherosclerosis Development

**DOI:** 10.1002/ptr.70391

**Published:** 2026-05-29

**Authors:** Lydia Symeonidi, Panagiota‐Efstathia Nikolaou, Panagiotis Stathopoulos, Andriana Christodoulou, Alexandra Svouraki, Panagiotis Efentakis, Christina Chania, Angeliki Choustoulaki, Maria Xenaki, Anna Agapaki, Maria Tsoumani, Nikolaos Kostomitsopoulos, Ioulia Tseti, Gemma Vilahur, Alexios‐Leandros Skaltsounis, Ioanna Andreadou

**Affiliations:** ^1^ Laboratory of Pharmacology, Department of Pharmacy National and Kapodistrian University of Athens Athens Greece; ^2^ Department of Clinical Therapeutics, Department of Medicine, School of Health Sciences National and Kapodistrian University of Athens Athens Greece; ^3^ Division of Pharmacognosy and Natural Products Chemistry, Department of Pharmacy National and Kapodistrian University of Athens Athens Greece; ^4^ Histochemistry Unit, Biomedical Research Foundation Academy of Athens (BRFAA) Athens Greece; ^5^ Centre of Clinical Experimental Surgery and Translational Research Biomedical Research Foundation of the Academy of Athens (BRFAA) Athens Greece; ^6^ Uni‐Pharma S.A. Athens Greece; ^7^ Research Institute Sant Pau, IIB‐Sant Pau, CIBERCV Barcelona Spain

**Keywords:** acute myocardial infarction, atherosclerosis, cardiometabolic syndrome, *Morus Alba*, *Vaccinium* sp.

## Abstract

Cardiovascular disease (CVD) remains the leading cause of morbidity and mortality worldwide. While studies suggest that berry fruits offer beneficial effects in CVD prevention, the bioactive compounds within different berries vary, and the specific mechanisms behind their protective effects are not yet fully elucidated. Our study aimed to evaluate and compare the cardioprotective and anti‐atherosclerotic effects of *Morus alba* (white mulberries, WMex) and *Vaccinium myrtillus* L. fruit extracts (blueberries, BBex), focusing on acute myocardial infarction (AMI), CVD risk factors, atherosclerosis development, and the underlying mechanisms. WMex and BBex were produced and their qualitative compositions were characterized. We evaluated the infarct size (IS) limiting potential of 8‐weeks supplementation with both WMex and BBex at nutritional doses in mice with western diet (WD)‐induced metabolic syndrome (MS). We further evaluated atherosclerosis development mitigation after 4‐weeks supplementation with the extracts in both male and female WD‐fed ApoE^−/−^ mice as well as the putative cardioprotective and atheroprotective mechanisms. The total flavonoids in WMex and BBex were 49.50 and 98.92 mg rutin/g extract, respectively, while the total phenolic content was 50.74 and 110.00 mg gallic acid/g extract, respectively. Both extracts limited IS compared to Control by suppressing apoptosis. WMex upregulated endothelial nitric oxide synthase (eNOS) phosphorylation and pathway along with circulating nitrite levels. WMex suppressed the extent of atheromatic area in both male and female ApoE^−/−^ mice, while BBex was effective in females without any of the extracts affecting estrogen levels. WMex downregulated NF‐kB‐ICAM‐1 axis in the atherosclerotic area and reduced circulating triglyceride levels. None of the extracts caused signs of toxicity, supporting a safety profile. In conclusion, WMex exerts potent cardioprotective and anti‐atherosclerotic properties in vivo and could be further exploited for CVD prevention.

## Graphical abstract

Schematic representation of the experimental design and the proposed mechanisms underlying the cardioprotective and anti‐atherosclerotic effects of Greek‐derived berry extracts. C57BL/6 mice with WD–induced MS and ApoE^−^/^−^ mice with atherosclerosis received chronic supplementation with WMex or BBex. In the myocardial ischemia–reperfusion model, both extracts reduced IS through anti‐apoptotic signaling. WMex activated the eNOS/NO/cGMP/PKG pathway. In the atherosclerosis model, WMex and BBex supplementation reduced atherosclerotic area. WMex downregulated NF‐κB signaling and ICAM‐1, while increasing eNOS activation. ApoE^**−/−**^: apolipoprotein E; BBex: blueberries extract; eNOS: endothelial‐nitric oxide synthase; ICAM‐1: intercellular adhesion molecule 1; IS: infarct size; MS: metabolic syndrome; NF‐κB: nuclear factor kappa B; NO‐cGMP‐PKG: nitric oxide‐cyclic guanosine monophosphate‐protein kinase G; WD: western diet; WMex: white mulberries extract.

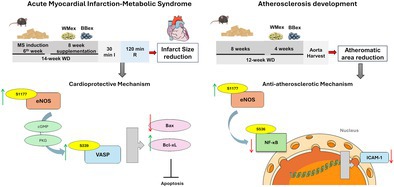

**Abbreviations**
Akt: protein kinase B
ALT: alanine aminotransferase
AMI: acute myocardial infarction
ANOVA: analysis of variance
AST: aspartic aminotransferase
Bcl‐xL: B‐cell lymphoma‐extra large
CVD: cardiovascular disease
DMSO: dimethyl‐sulfoxide
eNOS: endothelial‐nitric oxide synthase
GAPDH: glyceraldehyde‐3‐phosphate dehydrogenase
GSK‐3β: glycogen synthase kinase 3 beta
ICAM‐1: intercellular adhesion molecule 1
IL‐6: interleukin‐6
IRI: ischemia–reperfusion injury
IS: infarct size
LAD: left anterior descending artery
LDL: low‐density lipoprotein
MCP‐1: monocyte chemoattractant protein‐1
MS: metabolic syndrome
NF‐κB: nuclear factor kappa B
NLRP3: nucleotide‐binding oligomerization domain‐like receptor 3
NO‐cGMP‐PKG: nitric oxide‐cyclic guanosine monophosphate‐protein kinase G
p42/44 MAPK: P42/44 mitogen‐activated‐protein kinase
PBS: phosphate‐buffered saline
PCI: percutaneous intervention
RISK: reperfusion injury salvage kinase
ROS: reactive oxygen species
SAFE: survivor activating factor enhancement
SD: standard deviation
STAT3: signal transducer and activator of transcription 3
TTC: 2,3,5‐triphenyltetrazolium chloride
VASP: vasodilator‐stimulated phosphoprotein
VCAM‐1: vascular cell adhesion molecule‐1
WD: western diet

## 1 Introduction

Cardiovascular disease (CVD) is the leading cause of mortality and morbidity worldwide (Townsend et al. 2016). Acute myocardial infarction (AMI), the most common clinical manifestation of CVD, is treated primarily through primary percutaneous intervention (PCI) or thrombolytic therapy. Timely restoration of coronary flow is the cornerstone for minimizing infarct size (IS) and improving patient outcomes (Yellon and Hausenloy 2007). However, preventing complications and improving outcomes in ΑMI patients presents significant challenges. Key priorities include addressing atherosclerosis, as plaque erosion or rupture triggers thrombus formation (Vilahur and Fuster 2025) and developing strategies to effectively manage the damage caused by reperfusion, a condition referred to as myocardial ischemia/reperfusion injury (IRI) (Yellon and Hausenloy 2007). Strategies for reducing IS are essential alongside rapid reperfusion therapy. The implementation of pharmacological and mechanical interventions to mitigate IS has been unsuccessful in the clinical setting, highlighting the complex nature of IRI mechanisms (Ferdinandy et al. 2014). Suppressing IRI is mainly hindered by the presence of risk factors associated with metabolic syndrome (MS) including obesity, impaired glucose metabolism, insulin resistance, hypertension, and dyslipidemia (Silveira Rossi et al. 2022). These comorbidities interfere with the proposed mechanisms of cardioprotection and impair endothelial function.

Atherosclerosis is not only the primary cause of AMI, but it is also recognized as a low‐grade chronic inflammatory disease. This condition is characterized by increased endothelial permeability, allowing low‐density lipoprotein (LDL) to enter in the arterial intima, and facilitating monocyte infiltration. Endothelial cell activation further promotes monocytes recruitment by the expression of adhesion molecules such as vascular cell adhesion molecule‐1 (VCAM‐1), intercellular adhesion molecule‐1 (ICAM‐1) and chemokines. ΑMI has also been proposed to amplify inflammation in distant atherosclerotic plaques, since myocardial damage exacerbates pre‐existing chronic inflammation (Dutta et al. 2012).

Micronutrient supplementation represents a promising approach for addressing CVD risk factors and for improving the underlying mechanisms of IRI and atherosclerosis (An et al. 2022). Micronutrient supplementation may include phytochemicals, unsaturated fatty acids, antioxidants and minerals (An et al. 2022; Badimon et al. 2010). Berry extracts have shown beneficial effects on cardio‐metabolic risks, primarily due to their phytochemical compounds such as phenols and alkaloids. *Morus alba* fruit and its extracts have shown promise in mitigating MS parameters including obesity and hypertriglyceridemia in vivo (Chaiwong et al. 2021), while also exhibiting antioxidant properties (Chen et al. 2021). Similarly, *Vaccinium* sp. has anti‐atherosclerotic (Wu et al. 2010), antioxidant (Wu et al. 2010) and anti‐hypercholesteremic (Vendrame et al. 2013) effects in vivo. However, the cardioprotective potential of the aforementioned bioactive species against IRI, particularly in the presence of comorbidities, remains elusive.

With all this in mind, we aimed to investigate whether supplementation with berry extracts could exert multiple effects in MS, CVD, and atherosclerosis. At first, we sought to produce extracts from selected types of berries and blueberries grown in Greece, using green technology. The extract derived from White Mulberries ( *Morus alba* , WMex) and the extract from Blueberries (*Vaccinium* sp., BBex) were enriched in total flavonoids and total phenolic content. The aims of our study were to (1) characterize the cardioprotective potential of the extracts in terms of IS attenuation after chronic administration at nutritional doses in a murine in vivo model of MS and AMI, (2) decipher the underlying molecular mechanism of cardioprotection, (3) investigate the effects of the extracts on atheromatic area development in a murine in vivo model of atherosclerosis, and (4) provide mechanistic insights into their anti‐atherosclerotic mechanism. The protocols were designed to realize the preventive potential of berry extract supplementation in terms of cardioprotection and atheroprotection in the presence of comorbidities.

## 2 Materials and Methods

For complete methods and material provision, please refer to the Supporting Information.

### 2.1 Extracts Production and Qualitative Composition

WMex was obtained after processing natural‐dried fruits with acidified water and BBex was produced by processing freeze‐dried fruits with an acidified isopropanol/water mixture. In both cases, the removal of non‐active components (e.g., sugars, polysaccharides, polymers) and the enrichment of the extract with bioactives were carried out using absorption resin technology. Qualitative composition of extracts was performed with UPLC‐Orbitrap MS/MS and HPLC‐DAD Analysis.

### 2.2 Dose Selection

Two nutritional doses for WMex and BBex (250 and 500 mg/kg) were selected for chronic oral administration. These doses fall within the nutritionally relevant range commonly used for plant and berry‐derived extracts in rodent studies investigating metabolic and cardiovascular protection (Herrera‐Balandrano et al. 2021; Zhang et al. 2019).

### 2.3 Animals and Diet

In this study, 101 male and female C57BL/6 and 48 male and female ApoE^−/−^ mice, aged 8–10 weeks, were obtained from the Centre of Clinical Experimental Surgery and Translational Research, Biomedical Research Foundation Academy of Athens (BRFAA). Mice were housed in standard conditions (21°C–24°C and 40%–80% humidity), under a 12‐h light/dark cycle. Fresh water and either a standard chow diet or Western Diet (WD) supplemented with 1.25% cholesterol (Western Diet‐TD 88137, Ssniff) were provided ad libitum. All procedures conformed to the Presidential Decree 56/2013 for the Protection of Vertebrate Animals used for Experimental and other Scientific Purposes and the European Directive 2010/63/UE. The experimental protocol was approved by the competent Veterinary Service of the Prefecture of Athens (License protocol number: 893699/19‐09‐2022). Surgical procedures and interventions were performed according to the established guidelines (Lecour et al. 2021), that is, randomization was performed for all the cohorts of mice using randomization online tools, and the surgical procedures and the data analysis were performed by a blinded researcher to the respective treatments.

### 2.4 In Vivo Experimental Protocols

#### 2.4.1 First Experimental Protocol: Assessment of the Cardioprotective Effect of WMex and BBex in Mice With MS

**FIGURE 1:**
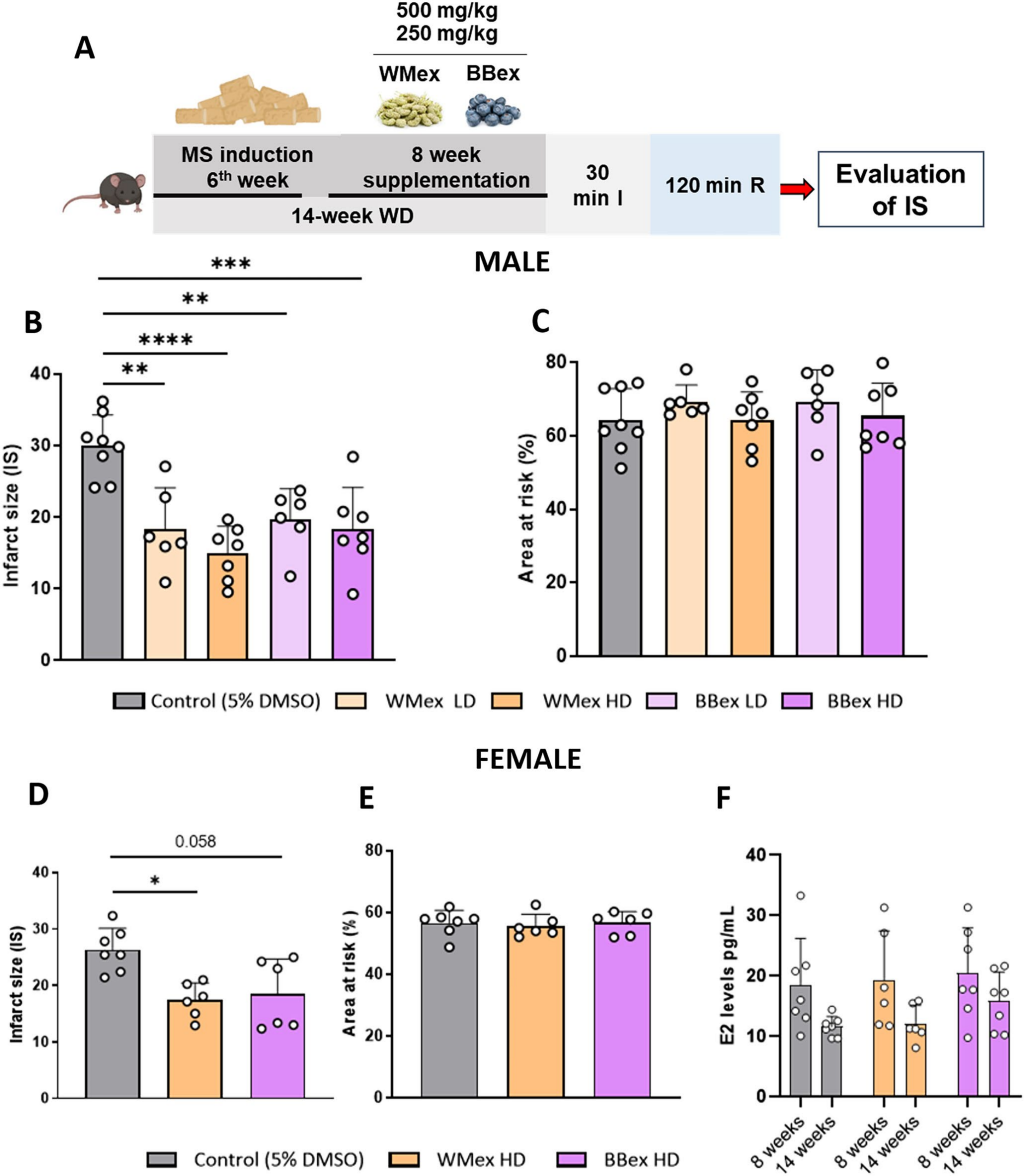
WMex and BBex supplementation reduces infarct size in both doses in male mice while only WMex HD reduces infarct size in female mice in the presence of MS comorbidities. (A) Schematic representation of the experimental protocol. Bar plots of (B) infarct size and (C) %area at risk in male mice. (D) Infarct size and (E) area at risk in female mice. Statistical significance was determined by one‐way ANOVA, Tukey's post hoc test for (B, E) and Kruskal–Wallis, Dunn's post hoc test for (C, D). (F) Estradiol (E2) circulating levels in female mice before and after the administration of the extracts. To determine the effect of the time, the extracts and their interaction, two‐way ANOVA analysis was performed. *p < 0.05, **p < 0.01, ***p < 0.001, ****p < 0.0001. The results are presented as means ± SD (*n* = 6–8). ANOVA: analysis of variance; BBex: blueberries extract; E2: estradiol; HD: high‐dose; LD: low‐dose; MS: metabolic syndrome; SD: standard deviation; WMex: white mulberries extract.

C57BL/6 mice were fed with WD for 14 weeks to induce MS. On week 6, mice were randomized into 5 groups (*n* = 6–8/group): 1. Control (5% DMSO), 2. WMex LD (250 mg/kg), 3. WMex HD (500 mg/kg), 4. BBex LD (250 mg/kg), 5. BBex HD (500 mg/kg) and supplemented daily for 8 consecutive weeks by oral gavage. Since the higher dose proved more effective in male mice, it was also administered to female mice (3 groups, *n* = 6–8/group). MS parameters (body weight, fasting glucose plasma levels and lipidemic profile) were assessed at baseline, 6th, and 14th week in all groups. Estradiol (E2) was determined before and after the administration of the extracts using the QuicKey Pro Mouse E2 (Estradiol) ELISA kit, CAT Number: E‐OSEL‐M0008 (Elabscience). The detection of the inflammatory biomarker interleukin‐6 (IL‐6) was assessed in mouse plasma on the 14th week using a commercial ELISA kit (OriGene Technologies), according to manufacturer's instructions. On week 14, mice were subjected to 30 min myocardial ischemia (I) followed by 120 min reperfusion (30′ I/120′ R) by left anterior descending artery (LAD) ligation. This protocol (Figure 1A) simulated the myocardial injury upon AMI and reperfusion therapy as we have previously reported (Castiello et al. 2025; Mylonas et al. 2025; Nikolaou et al. 2025). At the end of the reperfusion, IS was determined via Evans Blue/TTC staining to determine the cardioprotective effect of the extracts (Bøtker et al. 2018).

#### 2.4.2 Second Experimental Protocol: Investigation of the Underlying Molecular Mechanism of Cardioprotection Upon Supplementation With WMex and BBex in Mice With MS

**FIGURE 2:**
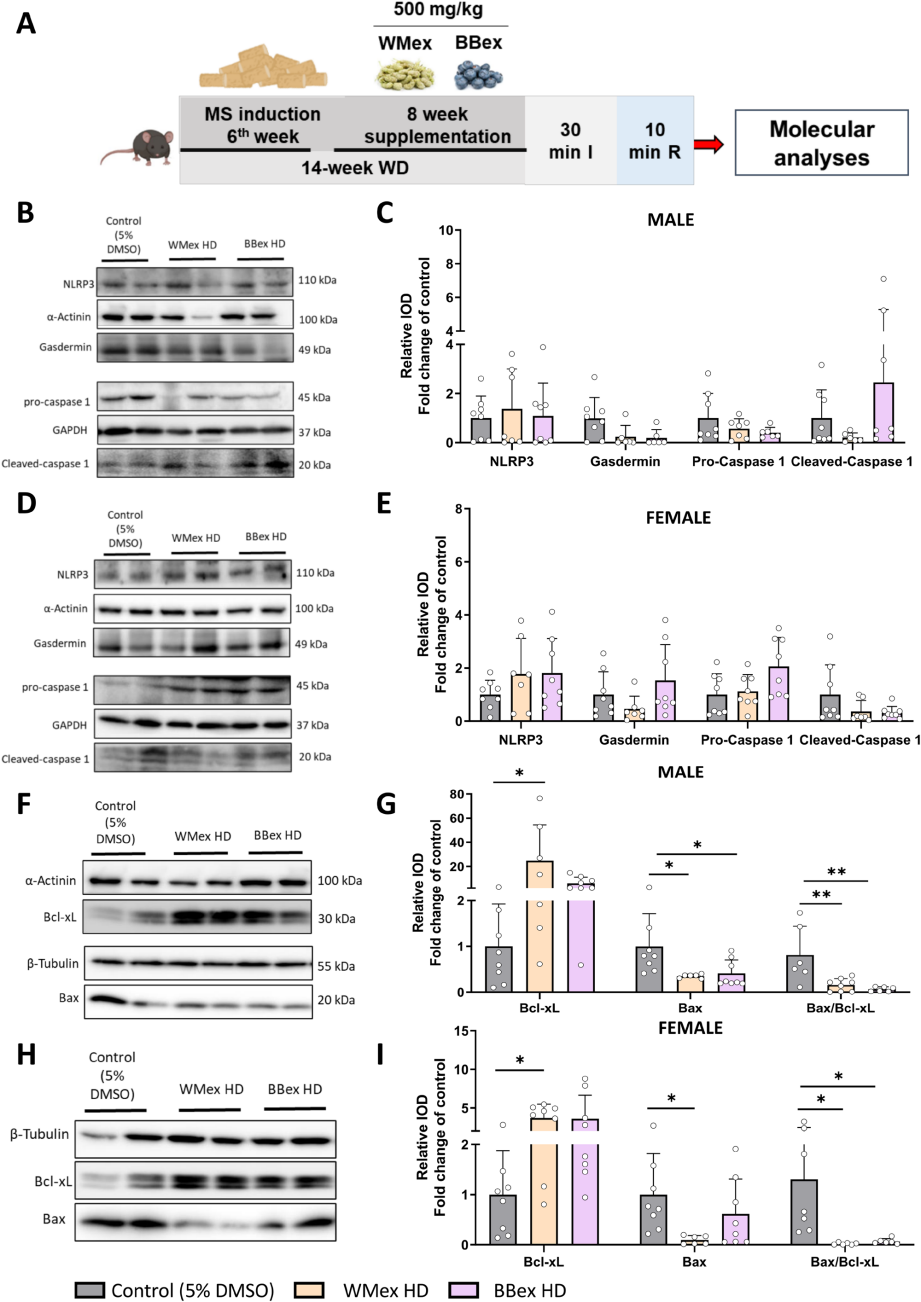
WMex and BBex supplementation reduces the apoptotic ratio of Bax/BcL‐xL in the reperfused myocardium of mice with MS. (A) Schematic representation of the experimental protocol. Representative western blots at the 10th min of reperfusion of NLRP3, a‐Actinin, Gasdermin, pro‐caspase 1, GAPDH, Cleaved‐caspase 1 in (B) male and (D) female mice. Relative densitometric graphs of NLRP3/α‐Actinin, Gasdermin/α‐Actinin, pro‐caspase 1/GAPDH, Cleaved‐caspase1/GAPDH in (C) male and (E) female mice. Representative western blots at the 10th min of reperfusion of a‐Actinin, Bcl‐xL, β‐Tubulin and Bax in (F) male and (H) female mice. Relative densitometric graphs of Bcl‐xL/a‐Actinin, Bax/β‐Tubulin and Bax/BcL‐xL ratio in (G) male mice and Bcl/xL/β‐Tubulin, Bax/β‐Tubulin and Bax/BcL‐xL ratio in (I) female mice. Statistical significance was determined by one‐way ANOVA, Tukey's post hoc test or Kruskal–Wallis, Dunn's post hoc test. *p < 0.05, **p < 0.01. The results are presented as means ± SD (*n* = 8). ANOVA: analysis of variance; BBex: blueberries extract; Bcl‐xL: B‐cell lymphoma‐extra synthase; MS: metabolic syndrome; GAPDH: glyceraldehyde‐3‐phosphate dehydrogenase; NPRP3: nucleotide‐binding oligomerization domain‐like receptor 3; WMex: white mulberries extract; SD: standard deviation.

To provide mechanistic insights on the observed IS reduction, supplementation with berry extracts was repeated as follows: in both sexes (*n* = 8/group): 1. Control (5% DMSO), 2. WMex (500 mg/kg), and 3. BBex (500 mg/kg). On week 14, mice were subjected to 30 min I followed by 10 min R (Figure 2A), the heart was excised, and the ischemic part was immediately frozen for protein analysis. This timepoint was selected based on previous reports regarding the early activation of cardioprotective mechanisms (Nikolaou et al. 2021, 2022, 2023). Circulating nitrite and nitrate levels were determined in mouse plasma using a commercially available kit (Cayman's Nitrate/Nitrite Colorimetric Assay Kit 780001), according to the manufacturer's instructions (Stamatelopoulos et al. 2019).

#### 2.4.3 Third Experimental Protocol: Characterization of the Anti‐Atherosclerotic Effect of WMex and BBex and Mechanistic Insights

Male and female ApoE^−/−^ mice were fed with WD for 12 weeks to accelerate atherosclerosis development (Christodoulou et al. 2024). On week 8, mice were randomized into 3 groups (*n* = 8/group): 1. Control (5% DMSO), 2. WMex (500 mg/kg), and 3. BBex (500 mg/kg), and the interventions were given daily for 4 consecutive weeks by oral gavage. The parameters of MS were also assessed in this model. On weeks 8 and 14, blood samples were collected for lipid level determination. Estradiol (E2) in mouse plasma was determined before and after the administration of the extracts using the QuicKey Pro Mouse E2 (Estradiol) ELISA kit, CAT Number: E‐OSEL‐M0008 (Elabscience). Mice were anesthetized with isoflurane (5% in L/min oxygen for induction), were euthanized via cervical dislocation, the descending thoracic aorta (including the distal aortic arch) was isolated, stained with Oil Red O, and atheromatic area was measured. The remaining aorta was perfused with cold PBS and immediately frozen for protein extraction and Western blot analysis (Figure 5A). The detection of the inflammatory biomarker IL‐6 was assessed in mouse plasma on the 12th week using a commercial ELISA kit (OriGene Technologies), according to manufacturer's instructions. Circulating nitrite and nitrate levels were determined using a commercially available kit (Cayman's Nitrate/Nitrite Colorimetric Assay Kit 780001), according to manufacturer's instructions (Stamatelopoulos et al. 2019).

#### 2.4.4 Forth Experimental Protocol: Toxicity Evaluation of Berry Extracts

The extracts were further tested for potential indications of toxicity. C57BL/6 mice were randomized into 3 groups (*n* = 7–8/group): 1. Control (5% DMSO), 2. WMex (500 mg/kg), and 3. BBex (500 mg/kg), and the interventions were given daily for 4 consecutive weeks by oral gavage. On week 4, blood samples were collected for the evaluation of hematological parameters as well as liver and kidney toxicity markers. Mice were anesthetized with isoflurane (5% in L/min oxygen for induction) and were euthanized via cervical dislocation.

### 2.5 Statistical Analysis

Statistical analysis was performed using GraphPad Prism 9.0.0 (Graph Pad Software Inc.) and data are presented as means ± standard deviation (mean ± SD). ROUT (*Q* = 1%) statistical test was used to identify outlier values which are excluded from analysis. All data were subjected to the Shapiro–Wilk and Kolmogorov–Smirnov normality test and were normally distributed unless otherwise stated. For Western blot densitometric data, MS induction, IS, lipid profile in mice with MS and atheromatic area, where three groups are compared, one‐way analysis of variance (ANOVA) was conducted, followed by Tukey's multiple comparisons test compared to control. For parameters not following normal distribution within groups, according to the Shapiro–Wilk and Kolmogorov–Smirnov normality test, the non‐parametric Kruskal–Wallis test was employed with Dunn's multiple comparisons post hoc test. Body weight and lipid profile in ApoE^−/−^ mice, where different time points are involved, were analyzed by two‐way ANOVA comparing the means among groups and using Tukey's multiple comparisons test compared to control as a post hoc test. Statistical significance was set at *p* < 0.05.

## 3 Results

### 3.1 Qualitative Extract Composition

LC‐HRMS and HPLC‐DAD chemical analyses showed that the WMex was rich in acids (quinic acid, malic acid, citric acid), phenolic acids (chlorogenic acid, 3‐p‐coumaroylquinic acid), pyrrolidine alkaloids (isomers of morusimic acids B, C, E), and flavonoids (rutin, quercetin, kaempferol, isoquercitrin, quercetin, as aglycones or with their corresponding glycosides). Meanwhile, in the BBex, the main secondary metabolites detected were anthocyanins (mainly delphinidin, petunidin, cyanidin and malvidin glycosides), acids (quinic acid, malic acid, citric acid), phenolic acids (gallic acid, caffeic acid hexoside, caffeic acid, chlorogenic acid, ferulic acid hexoside, ferulic acid, 3‐feruloylquinic acid), and flavonoids (rutin, isoquercitrin, myricetin, quercetin, kaempferol, quercitrin, gossypetin, isorhamnetin, syringetin, as aglycones or with their corresponding glycosides) (Figure S1–S6 and Tables S1–S4). The total flavonoids in the WMex and BBex were 49.50 and 98.92 mg rutin/g extract, while the total phenolic content was 50.74 and 110.00 mg gallic acid/g extract, respectively. Finally, the total monomeric anthocyanin content in the enriched BBex was 3.05% (expressed as Cyanidin 3‐glucoside).

### 3.2 Chronic Supplementation With WMex and BBex Reduces Myocardial IS in Mice With MS Without Altering MS‐Related Parameters

Since MS comorbidities affect the cardioprotective potential of several interventions (Ferdinandy et al. 2014), we investigated the IS‐limiting potential of chronic supplementation with WMex and BBex in mice with MS (Andreadou, Iliodromitis, et al. 2017).

First, we validated our model of MS. On week 6, MS induction was validated in male mice by a cluster of metabolic factors such as obesity, elevated blood glucose, and hypercholesterolemia. By week 14, these factors were either sustained or worsened, indicating MS establishment. Female mice demonstrated increased body weight and total cholesterol levels without an increase in blood glucose levels suggesting the absence of MS (Figure S7). On week 14, none of the extracts reversed the progression of obesity, lipid abnormalities, and hyperglycemia compared to control (5% DMSO) in either dose (Figure S8).

Then, we assessed the cardioprotective potential of the extracts. WMex and BBex supplementation significantly reduced IS in males at both doses compared to the control group (5% DMSO) (Figure 1B). Since the high dose resulted in a greater reduction in males, only the high dose was administered to females (Figure 1D). WMex HD exhibited the most potent cardioprotective effect in both sexes. No differences were observed in the area at risk among the different groups (Figure 1C,E). Overall, 5% DMSO has no effect on the investigated primary endpoints variables of the MS parameters or the ischemic insult (Christodoulou et al. 2024). To examine whether the cardioprotective effect in females is related to the levels of estrogens, we determined circulating 17‐β‐estradiol levels (E2) before and after the extract administration. Two‐way ANOVA suggested that E2 decreases over time which explained 22.34% of the total variation and showed a statistically significant effect (*p* = 0.0011) without significant differences among groups (Figure 1F). Therefore, WMex and BBex do not have estrogenic effects in the MS mice to interfere with their cardioprotective potential.

### 3.3 Cardioprotective Mechanism of WMex and BBex in Mice With MS

Myocardial IRI is mainly attributed to cardiomyocyte death, which can be divided into different types of cell death pathways including pyroptosis and apoptosis (Deng 2021). Regarding pyroptosis, we detected no significant changes in the expression of nucleotide‐binding oligomerization domain (NOD)‐like receptor protein 3 (NLRP3), Gasdermin, and Caspase‐1 in the ischemic myocardium compared to the control group, in either male (Figure 2B,C) or female mice (Figure 2D,E) at the 10th min of reperfusion. Thereafter, we focused on apoptosis and found a significant increase in the expression of the anti‐apoptotic mediator B‐cell lymphoma‐extra‐large (Bcl‐xL) after WMex supplementation compared to the control group in both sexes (Figure 2F–I). Furthermore, the expression of the apoptotic mediator Bax was significantly decreased by WMex supplementation compared to the control in both sexes, and by BBex only in male mice (Figure 2F,G) at the same timepoint (Castiello et al. 2025; Nikolaou et al. 2022). We also evaluated the apoptotic ratio, expressed as Bax/Bcl‐xL, to better understand the apoptotic/anti‐apoptotic balance in the reperfused myocardium (Castiello et al. 2025; Christodoulou et al. 2024). Our data showed a significantly lower Bax/BcL‐xL ratio following WMex and BBex supplementation in both sexes (Figure 2G,I).

**FIGURE 3:**
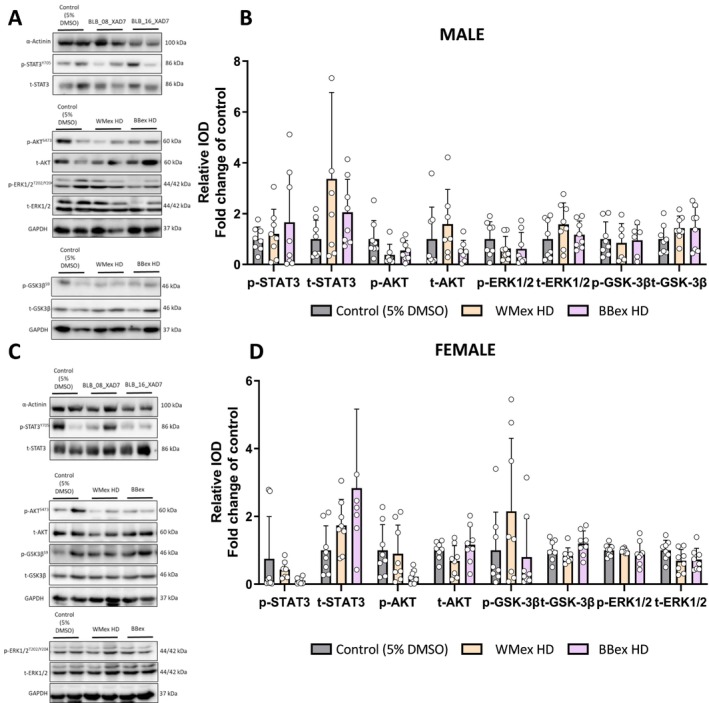
SAFE and RISK pathway are not activated upon supplementation with WMex and BBex extracts in mice with MS and AMI. Representative western blots at the 10th min of reperfusion of a‐Actinin, p‐STAT3 (Y705), t‐STAT3, p‐AKT (S473), t‐AKT, p‐ERK1/2 (T202/Y204), t‐ERK1/2, GAPDH, p‐GSK‐3β (S9), t‐GSK‐3β and GAPDH in (A) male and (C) female mice. Relative densitometric graphs of p‐STAT3 (Y705)/t‐STAT3, t‐STAT3/a‐Actinin, p‐AKT (S473)/t‐AKT, t‐AKT/GAPDH, p‐ERK1/2 (T202/Y204)/t‐ERK1/2, t‐ERK1/2/GAPDH, p‐GSK‐3β (S9)/t‐GSK‐3β, t‐GSK‐3β/GAPDH in (B) male and (D) female mice. Statistical significance was determined by one‐way ANOVA, Tukey's post hoc test or Kruskal–Wallis, Dunn's post hoc test. The results are presented as means ± SD (*n* = 8). AKT: protein kinase B; AMI: acute myocardial infarction; BBex: blueberries extract; ERK1/2: P42/44 mitogen‐activated‐protein kinase; GAPDH: glyceraldehyde‐3‐phosphate dehydrogenase; GSK‐3β: glycogen synthase kinase 3 beta; MS: metabolic syndrome; RISK: reperfusion injury salvage kinase; SAFE: survivor activating factor enhancement; SD: standard deviation; WMex: white mulberries extract.

We subsequently investigated whether the antiapoptotic effect could be attributed to known cardioprotective pathways activated early after reperfusion, such as the Reperfusion Injury Salvage Kinase (RISK) pathway and Survivor Activating Factor Enhancement (SAFE) (Yellon et al. 2023). Hence, we focused on investigating the phosphorylation and expression of protein kinase B (Akt; S473), p44/42 MAPK (Erk1/2; T202, Y204), Glycogen Synthase Kinase 3 beta (GKS‐3β; S9) and STAT3 (Y705) after 10 min of reperfusion. None of the extracts increased the phosphorylation or expression of these proteins in either male (Figure 3A,B) or female mice (Figure 3C,D), suggesting that cardioprotection is not due to RISK or SAFE pathway activation.

**FIGURE 4:**
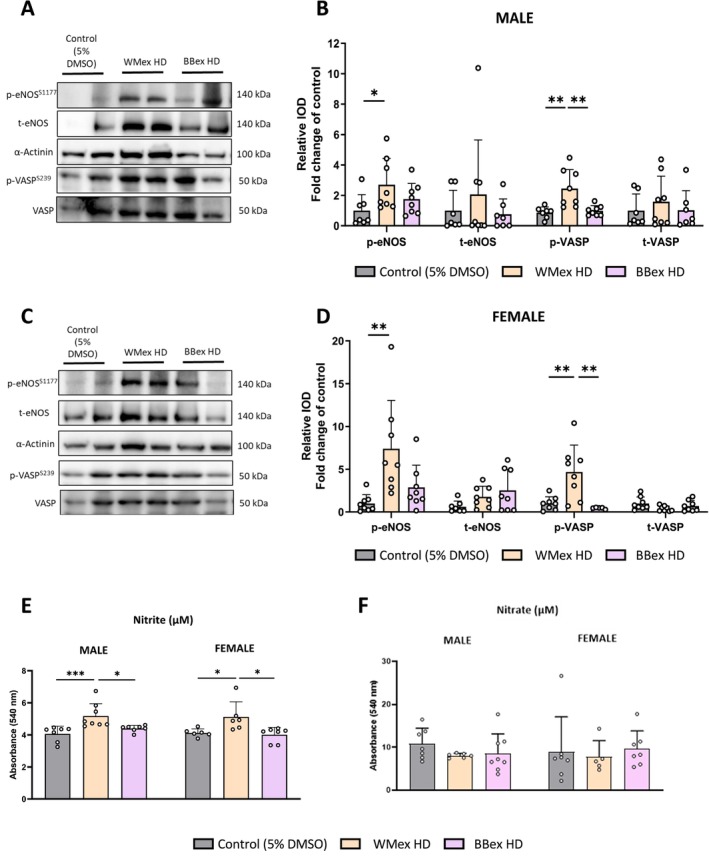
WMex supplementation upregulates eNOS and VASP phosphorylation in the reperfused myocardium of mice with MS. Representative western blots at the 10th min of reperfusion of p‐eNOS (S1177), t‐eNOS, α‐Actinin, p‐VASP (S239) and VASP in (A) male and (C) female mice. Relative densitometric graphs of p‐eNOS (S1177)/t‐eNOS, t‐eNOS/α‐Actinin, p‐VASP (S239)/VASP and VASP/α‐Actinin in (B) male and (D) female mice. (E) Nitrite circulating levels in male and female mice. (F) Nitrate circulating levels in male and female mice. Statistical significance was determined by one‐way ANOVA, Tukey's post hoc test or Kruskal–Wallis, Dunn's post hoc test. *p < 0.05, **p < 0.01. The results are presented as means ± SD (*n* = 8). ANOVA: analysis of variance; BBex: blueberries extract; eNOS: endothelial‐nitric oxide synthase; MS: metabolic syndrome; SD: standard deviation; VASP: vasodilator‐stimulated phosphoprotein; WMex: white mulberries extract.

Based on these findings, we hypothesized that other pro‐survival cardioprotective pathways might be involved in attenuating reperfusion injury such as the nitric oxide (NO)‐cyclic guanosine monophosphate (cGMP)‐protein kinase G (PKG) (Yellon et al. 2023). We detected a significant increase in endothelial nitric oxide synthase (eNOS) phosphorylation in both males (Figure 4A,B) and females (Figure 4C,D) supplemented with WMex. Furthermore, enhanced eNOS phosphorylation was associated with a subsequent increase in vasodilator‐stimulated phosphoprotein (VASP; S239), which is the major PKG phosphorylation site, also in male and female mice supplemented with WMex compared to the control group (Figure 4A–D), supporting the contribution of this pathway in WMex‐related IS‐limiting effects. Finally, we evaluated the nitrite/nitrate levels in mouse plasma to establish whether the cardioprotection is NO mediated. We determined a significant increase in nitrite levels in both male and female mice supplemented with WMex compared to the control group (Figure 4E), while nitrate levels remained unchanged (Figure 4F).

### 3.4 Chronic Supplementation With Berry Extracts Attenuates Atherosclerosis Development While Reducing Triglyceride Levels

**FIGURE 5:**
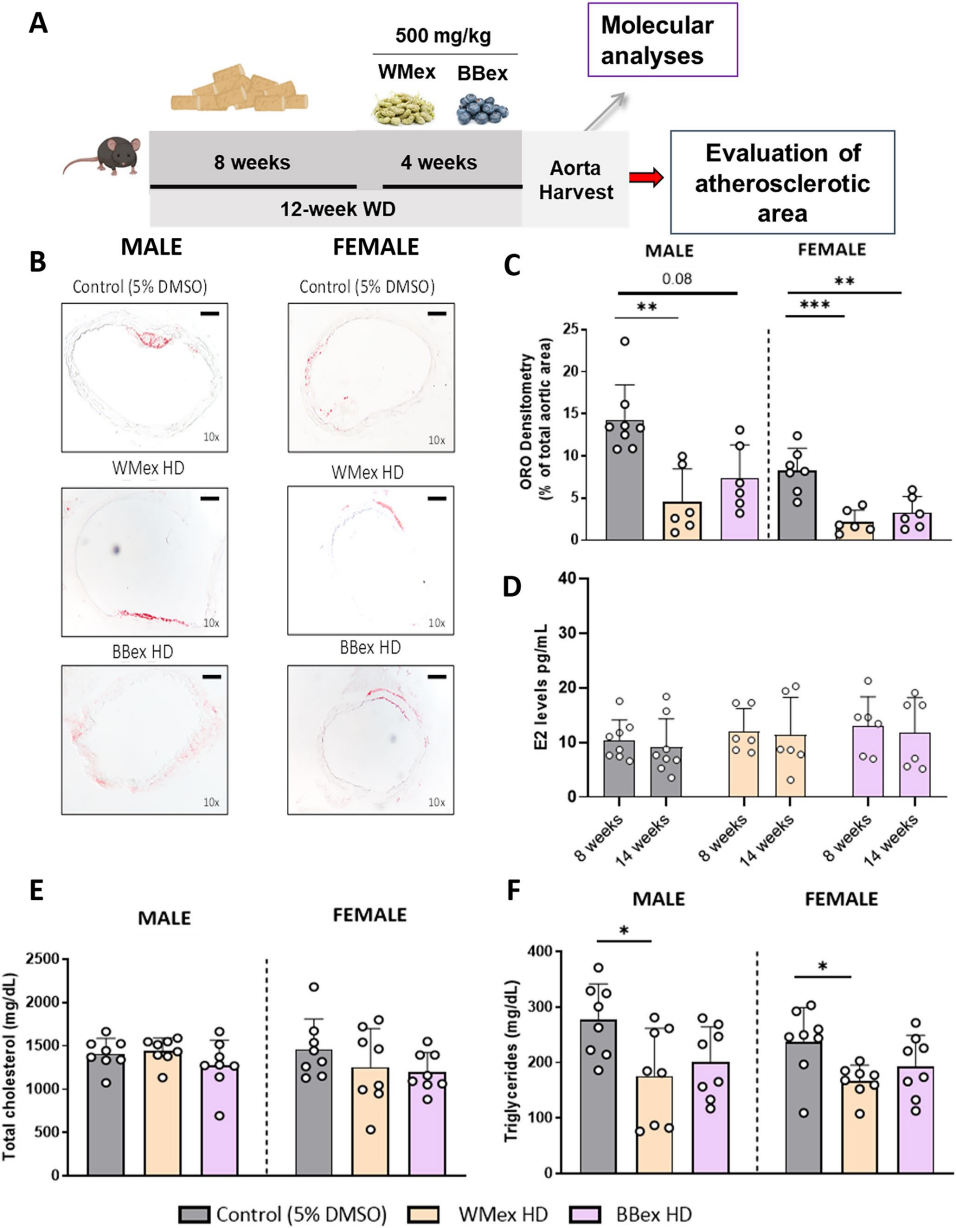
Anti‐atherosclerotic properties of WMex and BBex extracts after 4‐week supplementation in WD‐fed ApoE^−/−^ mice. (A) Schematic representation of the experimental protocol. (B) Representative images under brightfield microscope (scale: 150 μm) from ApoE^−/−^ aortic sections. (C) Oil Red O (ORO) densitometry (% total aortic area) in aortic rings of ApoE^−/−^ mice. (D) Estradiol (E2) circulating levels in female mice before and after the administration of the extracts. (E) Circulating total cholesterol levels (mg/mL). (F) Circulating triglyceride levels (mg/mL). Statistical significance was determined by one‐way ANOVA, Tukey's post hoc test for (C) (female), (E, F) and Kruskal–Wallis post hoc test for (C) (male). In (D), to determine the effect of the time, the extracts and their interaction, two‐way ANOVA analysis was performed. *p < 0.05, **p < 0.01, ***p < 0.001. The results are presented as means ± SD (*n* = 6–8). ANOVA: analysis of variance; ApoE^−/−^: apolipoprotein E; BBex: blueberries extract; E2: estradiol; SD: standard deviation; WD: western diet; WMex: white mulberries extract.

Since MI and atherosclerosis are tightly interconnected processes (Aboonabi et al. 2019), we hypothesized that oral supplementation with berry extracts would render beneficial effects on atherosclerosis development. WD‐fed ApoE^−/−^ mice, besides baring atherosclerosis, had increased body weight, fasting glucose levels, and total cholesterol levels, supporting that MS was also induced in this model in both sexes (Figure S9). WMex supplementation significantly reduced atherosclerotic area by Oil Red O staining, indicating anti‐atherosclerotic properties in both male and female mice. BBex supplementation significantly decreased atherosclerotic area in female mice, and there was a trend toward reduction in males (Figure 5B,C). To examine whether the atheroprotective effect in females is related to the levels of estrogens, we determined circulating E2 levels before and after the extract administration, but no significant differences were detected (Figure 1D) suggesting that neither WMex nor BBex have estrogenic potential that interferes with atheromatic area development. A significant decrease in triglyceride levels by WMex was observed while circulating total cholesterol levels remained unchanged (Figure 5E,F) implying that the mechanism of atherosclerosis mitigation by both extracts cannot be solely attributed to improvements in the lipid profile. Body weight, high‐density lipoprotein (HDL), and LDL levels remained unaffected by WMex and BBex supplementation (Figure S10).

### 3.5 Chronic Supplementation With WMex Increased eNOS Activation and Decreased NF‐kB‐ICAM‐1 Axis in the Aortic Tissue

**FIGURE 6:**
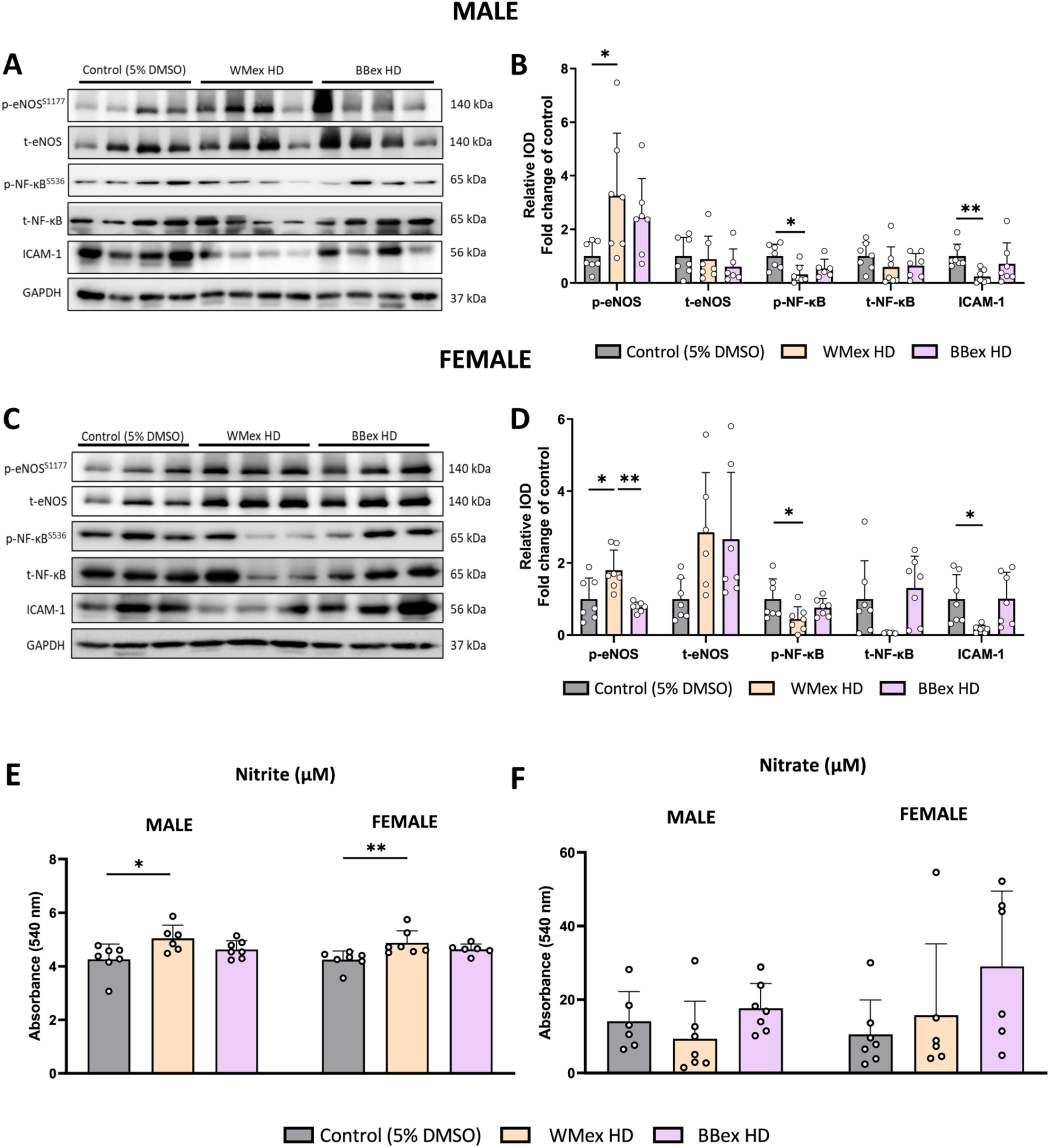
WMex supplementation downregulates NF‐κB phosphorylation while increasing eNOS phosphorylation in aortas of WD‐fed ApoE^−^/^−^ mice. Representative western blots of p‐eNOS (S1177), t‐eNOS, p‐NF‐κB (S536), t‐ NF‐κB, ICAM‐1 and GAPDH in (A) male and (C) female mice. Relative densitometric graphs of p‐e‐NOS (S1177)/t‐eNOS, t‐eNOS/GAPDH, p‐NF‐κB (S536)/t‐NF‐κB, t‐NF‐κB/GAPDH and ICAM‐1/GAPDH in (B) male and (D) female mice. (E) Nitrite circulating levels in male and female mice. (F) Nitrate circulating levels in male and female mice. Statistical significance was determined by one‐way ANOVA, Tukey's post hoc test or Kruskal–Wallis, Dunn's post hoc test. *p < 0.05, **p < 0.01. The results are presented as means ± SD (*n* = 7). ANOVA: analysis of variance; ApoE^**−/−**^: apolipoprotein E; eNOS: endothelial‐nitric oxide synthase; GAPDH: glyceraldehyde‐3‐phosphate dehydrogenase; ICAM‐1: intercellular adhesion molecule 1; NF‐κB: nuclear factor kappa B; SD: standard deviation; WD: western diet; WMex: white mulberries extract.

Since atherosclerosis is a chronic inflammatory disease (Miki et al. 2012), closely linked to endothelial dysfunction (Querio et al. 2023), we further investigated the potential anti‐atherosclerotic mechanism of the extracts, focusing on the nuclear factor kappa B (NF‐κB) pathway and eNOS activation. WMex supplementation led to a significant reduction in the phosphorylation levels of NF‐κB and in intercellular adhesion molecule‐1 (ICAM‐1) levels in the aorta in both sexes. Furthermore, we observed a significant increase in e‐NOS activation (phosphorylation at S1177) (Figure 6A–D). Additionally, we evaluated the nitrite and nitrate circulating levels, and we found that WMex increased circulating nitrite levels in both sexes in agreement with eNOS phosphorylation in the aortic rings (Figure 6E), while nitrate levels remained unchanged (Figure 6F).

Finally, since MS comorbidities, AMI and atherosclerosis are processes that are linked to systemic inflammation (Andreadou et al. 2025; Libby et al. 2002), we determined IL‐6 levels in both mice with MS and ApoE^−/−^ mice in order to gain insights on the putative systemic anti‐inflammatory effect of the extracts. No significant changes were observed in circulating IL‐6 levels in either male or female mice (Figure S11).

### 3.6 Toxicity Evaluation of Berry Extracts In Vivo

The potential subacute toxicity of the extracts was examined at the selected dose for 4 weeks. None of the extracts caused significant changes in toxicity or hematologic parameters compared to the control group (5% DMSO), supporting a safety profile (Tables 5 and 6).

## 4 Discussion

In the present study, we produced and characterized the cardioprotective and anti‐atherosclerotic properties of Greek‐derived berry‐based extracts with the aim to suggest a nutritional supplementation for primary and secondary CVD prevention. The protocols were designed to realize the preventive potential of berry extract supplementation in terms of cardioprotection and atheroprotection in the presence of pre‐existing comorbidities. The administration of the berry extracts was performed after the establishment of the MS to mimic the pathophysiology of hyperlipidemia, diabetes, and obesity. Therefore, the curative effect of the extracts against MS was examined. Then, the administration begins, which is before the ischemic insult of AMI or before the establishment of atherosclerotic plaques. In this way, the preventive potential against acute myocardial injury and atherosclerosis is examined. Our data reveal that none of the extracts alter the parameters of hyperlipidemia, diabetes, and obesity. In WD‐induced MS mice model, both extracts limited myocardial IS in males, with WMex also being cardioprotective in females. We further demonstrated that IS limitation by the berry extracts is mediated via the activation of the pro‐survival NO/cGMP/PKG pathway, also indicative by the increase in circulating nitrite levels and reduced apoptosis. Moreover, WMex supplementation also reduced aortic atherosclerosis development in both sexes, concomitantly with the upregulation of eNOS phosphorylation and nitrites bioavailability. Atheroprotection upon BBex supplementation was less pronounced and observed only in female mice. Finally, WMex intake reduced triglyceride levels, and no safety issues were reported for both berry extracts. Overall, our study proposes a potential nutritional intervention based on WMex with proven cardiovascular protective efficacy and safety profile. Notably, to the best of our knowledge, the properties of white mulberry extracts remain largely understudied, underscoring the novelty and significance of our findings.

MS favors atherosclerosis progression, leading to CVD manifestations such as MI (Miki et al. 2012). To enhance the translational value of our study, we firstly assessed the cardioprotective potential of berry extracts in animals with WD‐induced MS, a condition known to impair the endogenous cardioprotective signaling pathways hampering the effectiveness of multiple cardioprotective interventions (Andreadou et al. 2020; Lecour et al. 2021). Hence, we used a previously described WD‐induced MS model, characterized by the development of obesity, hypercholesterolemia and hyperglycemia, emerging by the 6th week of WD administration (Christodoulou et al. 2024). Female mice had increased body weight and total cholesterol levels by week 6 of WD administration but normal glucose levels indicating that they did not develop MS. This resistance to the development of typical MS symptoms has been previously described (Pettersson et al. 2012). Berry extract administration began once the MS comorbidities were established, thereby enhancing the translatability potential of our approach. Of note, intake of the extract exerted no effects on body weight, blood glucose levels and lipid profile in mice with MS. Interestingly, IS was significantly reduced by the berry extracts at all tested doses in male mice in the presence of MS indicating that the cardioprotective effect pertains in the presence of comorbidities. Our findings align with our previous study which demonstrated cardioprotection following pretreatment with other natural compounds (Christodoulou et al. 2024), and herein we highlight for the first time the IS‐liming properties of the Greek‐based berry extracts. Importantly, we report that as the model of MS progresses from the 8th to 14th week, the circulating levels of E2 are significantly decreased over time, highlighting the complex relationship between estrogens and MS comorbidities (Lizcano and Guzmán 2014; Xiao and Liu 2024). The administration of the extracts did not prevent E2 decrease indicating that the extracts do not possess estrogenic effects and their cardioprotective benefits do not relate to the E2 levels.

To identify the cardioprotective mechanism behind, we firstly focused on pyroptosis, which is a proinflammatory programmed cell death type (Deng 2021). However, we did not observe any effect on the expression of NLRP3, Gasdermin or Caspase‐1. Thereafter, we focused on apoptosis since it is activated during the early stages of reperfusion (Castiello et al. 2025; Nikolaou et al. 2022). Both WMex and BBex resulted in a decreased apoptotic Bax/Bcl‐xL ratio supporting their ability to prevent apoptotic‐related cell death (Nikolaou et al. 2022). Although, activation of the RISK and SAFE pathway has been linked to the inhibition of apoptotic signaling (Yellon et al. 2023), we observed no impact on the key‐mediators GSK‐3β, Akt or STAT3 (Yellon et al. 2023). Yet, WMex administration was associated with the activation of the NO/cGMP/PKG pathway, which is of paramount importance in the survival signaling in the reperfused myocardium (Burley et al. 2007). NO is produced by eNOS and has notable protective effects, such as preventing platelet aggregation by activating soluble guanylate cyclase (sGC) in platelets and supporting cardiac function and smooth muscle relaxation via the eNOS/cGMP/PKG signaling pathway, which reduces intracellular calcium levels (Nauli 2022). NO also exhibits antioxidant properties by upregulating heme oxygenase‐1 and ferritin, reducing superoxide anion levels (Nauli 2022). Activation of eNOS further corroborated with VASP S239 phosphorylation suggesting downstream PKG activation. PKG activation has been extensively associated with cardioprotection in terms of IS limitation (Inserte and Garcia‐Dorado 2015). To further support the involvement of NO signaling, we measured plasma nitrite and nitrate levels to assess whether the observed cardioprotection was mediated through NO‐derived circulating metabolites. Indeed, WMex supplementation led to a significant increase in the circulating nitrite levels in mice with MS, further supporting the involvement of NO‐dependent mechanisms in the observed cardioprotection. Emerging evidence suggests that eNOS‐derived NO may mediate not only local endothelial effects but also endocrine actions, largely through the systemic transport of nitrite and other semistable NO metabolites (Leo et al. 2021). The eNOS phosphorylation and activation corroborates with nitrite levels in several settings of cardioprotection and atheroprotetion in agreement with our findings (Bradley et al. 2015; Bryan et al. 2008; Nguyen et al. 2023; Sharma et al. 2015). Finally, WMex supplementation conferred significant cardioprotection even in the presence of metabolic comorbidities. The absence of changes in body weight, lipid profile, or glycemia indicates that IS reduction is primarily attributed to local cytoprotective signaling rather than to cardiometabolic improvements, which involve direct myocardial and endothelial mechanisms of the eNOS/NO/cGMP/PKG signaling pathway and reduced apoptotic protein ratio. BBex led to a marginal insignificant reduction in IS in female mice, which questions its translation in both sexes in humans and highlights WMEx as more efficient cardioprotective strategy for future studies. The effect on eNOS phosphorylation could be either indirect via upstream modulation or even systemic by restoring NO signaling. Previous reports suggest that *Moruls alba* extract alleviates hypertension and this systemic effect was in parallel with eNOS phorsphorylation (Carrizzo et al. 2016). In fact, the authors also suggest that the activation of eNOS may involve additional proteins including the enhanced phosphorylation of PERK at threonine^980^ and HSP90 at threonine^5/7^ (Carrizzo et al. 2016). Additionally, improvement in vascular remodeling by *Morus alba* extract has been observed in rats via preventing smooth muscle cell proliferation, thickening of the tunica media, and vascular hyper‐reactivity (Park et al. 2018). These effects could explain systemic alleviation of the vascular wall and therefore indirect effects on eNOS signaling. Therefore, several studies support that the observed modulation of eNOS signaling is likely attributable to the synergistic effects of the mulberry extract's constituents, which is consistent with our in vivo findings (Carrizzo et al. 2016; Park et al. 2018).

We further investigated the effect of berry extracts in an in vivo model of atherosclerosis. WMex supplementation reduced atheromatic area development in both male and female mice, while BBex was effective in female ApoE^−/−^ mice. Thereafter, we evaluated the potential anti‐atherosclerotic mechanism of action. Chronic inflammation is directly involved in the increase of NF‐κB signaling. During atherosclerosis development, NF‐κB upregulation in endothelial cells leads to endothelial dysfunction with the consequent reduction of eNOS expression and activation and NO release. NF‐κB signaling, in turn, induces the upregulation of ICAM‐1, which is linked to atherosclerosis development (Querio et al. 2023). Our data revealed a suppression of NF‐κΒ phosphorylation and ICAM‐1 expression in aortic tissue of both male and female WMex‐supplemented mice. Furthermore, these findings were accompanied by a significant increase in e‐NOS activation. The findings related to NF‐κB signaling align with an in vitro study showing that the treatment with an ethanol extract of white mulberry fruit reduced the activation of LPS‐stimulated RAW 264.7 macrophages, suggesting its anti‐inflammatory potential (Yu et al. 2021). Overall, the observed protective effects of the WMex against CVD manifestations in this study are associated with the upregulation of eNOS phosphorylation.

Finally, WMex supplementation induced a reduction in circulating triglycerides in ApoE^−/−^ mice, without affecting total cholesterol levels, highlighting a novel lipid‐modulating action with potential therapeutic relevance for hypertriglyceridemia, not previously reported (Wani et al. 2023). Previous studies suggest that triglycerides are independent predictors of endothelial function, closely associated with atherosclerosis (Kajikawa et al. 2016). It has been reported that elevated triglyceride levels contribute to adverse cardiovascular outcomes, while reducing circulating triglyceride levels has been associated with endothelial function improvement (Kajikawa et al. 2016). Several studies indicate that mulberry extracts containing alkaloids, polyphenols, and flavonoids have antioxidant and lipid‐lowering effects and can reduce not only serum lipid levels but also atheromatic area development, thus improving atherosclerosis. Furthermore, mulberry leaf tablet therapy in patients with mild dyslipidemia improved serum lipid levels, suggesting that its beneficial effects may be related to the regulation of hepatic expression of PPARγ target genes, which are involved in lipid and lipoprotein metabolism. On the other hand, mulberry leaves are rich in alkaloids, a potent α‐glucosidase inhibitor, which lowers plasma glucose levels and subsequently reduces fatty acid influx to the liver, contributing to decreased triglyceride and cholesterol levels. In addition, flavonoids inhibit hydroxy‐3‐methylglutaryl CoA reductase, a mechanism similar to statin lipid‐lowering drugs. Therefore, mulberry extracts contain constituents that can act synergistically and can modulate lipid metabolism via complex mechanisms, which is in accordance with our findings (Aramwit et al. 2011; Chan et al. 2013; Lupo et al. 2019; Kobayashi et al. 2016). However, the mechanism by which WMex reduces circulating triglycerides remains to be further investigated. Although atherosclerosis is closely linked with chronic systemic inflammation (Ajoolabady et al. 2024), IL‐6 plasma levels remained unchanged following chronic supplementation with WMex or BBex extracts, suggesting that circulating IL‐6 modulation is not linked to the berry extracts' atheroprotective potential. The atheroprotective effect of WMex is more pronounced than BBex and it is attributed to the activation of eNOS and downregulation of NFkB‐ICAM‐1 pathway in the aortic rings without systemic alterations in inflammatory markers such as IL‐6. As it has been previously reported in the literature, only a part of the patients with cardiovascular diseases have pronounced systemic inflammation and could benefit from anti‐inflammatory treatments (Andreadou et al. 2025). Therefore, the lack of effect of the extracts on systemic IL‐6 levels does not limit the translational applicability of WMex as an atheroprotective strategy. The safety profile of the extracts was further evaluated in a subacute toxicity study. Daily oral administration of WMex and BBex (250 and 500 mg/kg) for 4 weeks in C57BL/6 mice did not produce any significant changes in hematological parameters or serum markers of liver and kidney function compared to control animals (5% DMSO). These findings support the absence of overt systemic or organ‐specific toxicity at the doses used in the efficacy studies and provide preliminary evidence that both extracts are safe for nutritional use at the tested doses.

Regarding the translation of our findings in humans, several points should be considered including the dose and the relationship between effect sizes and clinical outcomes. Upon converting the animal dose to the Human Equivalent Dose (HED) according to Nair and Jacob dose convention paper (Nair and Jacob 2016), the effective dose of berry extracts corresponds to a daily intake of approximately 1.2–2.4 g, which can be formulated as an oral supplementation. The doses selected (250 and 500 mg/kg) are within the nutritionally relevant range commonly used for plant and berry‐derived extracts in rodent studies investigating metabolic and cardiovascular protection (Herrera‐Balandrano et al. 2021; Zhang et al. 2019). The observed benefits by the berry extracts in our studies were the reduction in IS by 5%–15% and the reduction by 5%–10% of oil red O positive area which have been proven to translate into patient outcome improvement. Specifically, a clear graded association has emerged between IS and adverse outcomes in patients. For every 5% increase in IS, the risk of mortality increased substantially, and a similar pattern was observed for hospitalization due to heart failure (Stone et al. 2016). These relationships remained robust after adjustment for major clinical and procedural factors, including age, sex, diabetes, hypertension, hyperlipidemia and smoking status, among others (Stone et al. 2016). These data indicate that nutraceutical interventions relieving the ischemic insult can lead to meaningful translation. Regarding atherosclerosis, the use of statins has been effectively proven to prevent atherosclerosis progression, improve plaque composition and has revolutionized patient outcomes (Almeida and Budoff 2019; Park et al. 2016). Therefore, nutraceutical supplementation in specific populations at risk could be envisioned on top of the standard of care.

Despite our aim to provide high translational value in our in vivo protocols and evaluate both sexes, certain limitations deserve to be acknowledged. The major limitation of the study is that it does not provide causal relationships between the dominant extract constituents and the cardioprotective or atheroprotective mechanisms since the primary goal of the study was to evaluate the berry extracts as nutraceuticals. In the present study, we found the presence of multiple compounds, phenolics, flavonoids, and anthocyanins; however, the identification of the key bioactive compounds responsible for the observed effects remains to be elucidated. Therefore, we hypothesized that the beneficial effects of the extracts are likely attributed to the synergistic effect of all the above. Specifically, BBex contains anthocyanins, which are established antioxidants with cardioprotective benefits (Aboonabi et al. 2019; Cassidy et al. 2016; Festa et al. 2023; Garcia and Blesso 2021; Mohammadi et al. 2024), possibly accounting for the increased myocardial salvage and reduced atheromatic area. WMex contains pyrrolidine alkaloids, which are underexplored with several pharmacological effects that have been noted (Islam and Mubarak 2020). Extensive research on their pharmacological targets is ensured for the future. Differences in atheromatic area were noted, with female mice developing smaller lesions than males in accordance with previous reports (Zhang et al. 2018). WD did not successfully induce MS in female mice, likely due to their increased resistance to the manifestations of MS (Pettersson et al. 2012). Finally, we provide safety data for the administration of the extracts for 1 month; however, long‐term hepatic and renal toxicity cannot be ruled out based on the presented data. The validity of the experimental models and the efficacy of established interventions have already been reviewed (Andreadou et al. 2020) and experimentally demonstrated in previous work from our laboratory, thereby alleviating the need for a positive control in this context (Andreadou, Efentakis, et al. 2017; Christodoulou et al. 2024).

## 5 Conclusion

Herein, our study demonstrates that Greek‐based berry extracts can be used as a nutritional intervention to provide protective properties against IRI and atherosclerosis development. Particularly, our study revealed that the most potent extract is WMex which exerts both direct cardioprotective effects, anti‐atherosclerotic properties and mitigated hypertriglyceridemia, highlighting the favorable pharmacological properties of white mulberries.

Notably, given that white mulberry extracts remain largely understudied, our work provides novel insights and paves the way for future research on bioactive constituents such as pyrrolidine alkaloids (e.g., morusimic acids), which may represent promising therapeutic agents against metabolic comorbidities and AMI (Bouillon and Meyer 2007; Chaiwong et al. 2021).

## Author Contributions

**Gemma Vilahur:** writing – review and editing, resources, supervision. **Ioulia Tseti:** resources, project administration, funding acquisition. **Andriana Christodoulou:** methodology, investigation. **Alexios‐Leandros Skaltsounis:** resources, writing – review and editing, project administration, supervision. **Panagiotis Efentakis:** methodology, investigation. **Alexandra Svouraki:** investigation. **Ioanna Andreadou:** writing – review and editing, project administration, supervision, funding acquisition, conceptualization. **Nikolaos Kostomitsopoulos:** writing – review and editing, resources. **Christina Chania:** investigation. **Angeliki Choustoulaki:** investigation. **Maria Xenaki:** investigation. **Anna Agapaki:** resources, investigation. **Panagiotis Stathopoulos:** writing – review and editing, methodology, investigation, formal analysis. **Panagiota‐Efstathia Nikolaou:** writing – review and editing, visualization, methodology, investigation, conceptualization, supervision. **Lydia Symeonidi:** writing – original draft, methodology, investigation, formal analysis. **Maria Tsoumani:** writing – review and editing, investigation, conceptualization.

## Funding

This work was supported by the Operational Program “Competitiveness, Entrepreneurship and Innovation” of the General Secretariat for Research and Innovation, Hellenic Republic Ministry of Development, Greece under the call “RESEARCH—CREATE—INNOVATE” (project code: 03427).

## Conflicts of Interest

The authors whose names are listed immediately below report their involvement in the patent with Application #No. GR20240100388 and Grant #No. GR1011012, entitled “Composition based on berries extract suitable for cardioprotection.” Author names: Ioanna Andreadou, Alexios‐Leandros Skaltsounis, Ioulia Tseti, Lydia Symeonidi, Panagiotis Stathopoulos, and Andriana Christodoulou. The other authors declare no conflicts of interest.

## Supporting information


**Data S1:** Supplementary methods.
**Figure S1:** LC‐HRMS chromatogram, recorded in positive ion mode, of the WMex. The identified bioactive compounds are highlighted.
**Figure S2:** LC‐HRMS chromatogram, recorded in negative mode, of the WMex. The identified bioactive compounds are highlighted.
**Figure S3:** LC‐HRMS chromatogram recorded, in positive ion mode, of the BBex. The identified bioactive compounds are highlighted.
**Figure S4:** LC‐HRMS chromatogram, recorded in negative ion mode, of the BBex. The identified bioactive compounds are highlighted.
**Figure S5:** HPLC‐DAD chromatogram, recorded at 525 nm of the BBex.
**Figure S6:** HPLC‐DAD chromatogram, recorded at 525 nm of the WMex.
**Figure S7:** Assessment of MS induction in C57Bl6 mice fed on Western diet (WD) with a cluster of metabolic factors such as body weight, blood glucose levels, total cholesterol and triglycerides. (A) Effect of WD on body weight in male and female mice. (B) Effect of WD on blood glucose levels in both sexes and (C) effect of WD on total cholesterol and triglyceride levels in both sexes. Statistical significance was determined by one‐way ANOVA, Tukey's post hoc test. *p < 0.05, **p < 0.01, ***p < 0.001, ****p < 0.0001. The results are presented as means ± SD (*n* = 8). ANOVA: analysis of variance; MS: metabolic syndrome; SD: standard deviation.
**Figure S8:** Effect of 8‐week supplementation with berry extracts on body weight and blood glucose levels in Western diet (WD) fed C57Bl6 mice. (A) Body weight in male mice. (B) Blood glucose levels in male mice. (C) Lipid profile in male mice. (D) Body weight in female mice. (E) Blood glucose levels in female mice and (F) lipid profile in female mice. Statistical significance was determined by one‐way ANOVA, Tukey's post hoc test for (B–F) and Kruskal–Wallis, Dunn's post hoc test, for (A). The results are presented as means ± SD (*n* = 6–8). ANOVA: analysis of variance; SD: standard deviation.
**Figure S9:** Assessment of MS induction with a cluster of metabolic factors such as body weight, blood glucose, total cholesterol and triglycerides in Western diet (WD) fed ApoE^(−/−)^ mice. (A) Effect of WD on body weight in male and female mice. (B) Effect of WD on blood glucose levels in both sexes and (C) effect of WD on total cholesterol and triglyceride levels in both sexes. Statistical significance was determined by one‐way ANOVA, Tukey's post hoc test. *p < 0.05, **p < 0.01, ***p < 0.001, ****p < 0.0001. The results are presented as means ± SD (*n* = 8). ApoE^−/−^: apolipoprotein E; ANOVA: analysis of variance; MS: metabolic syndrome; SD: standard deviation.
**Figure S10:** Effect of 4‐week supplementation with berry extracts on body weight and lipid levels in Western diet (WD) fed ApoE^(−/−)^ mice. (A) Body weight in both sexes, (B) HDL levels in both sexes and (C) LDL levels in both sexes. Statistical significance was determined by two‐way ANOVA, Tukey's post hoc test. The results are presented as means ± SD (*n* = 8). ANOVA: analysis of variance; ApoE^−/−^: apolipoprotein E; HDL: high‐density lipoprotein; LDL: low‐density lipoprotein; SD: standard deviation.
**Figure S11:** Effect of IL‐6 inflammatory biomarker in both mice with MS and ApoE^(−/−)^. (A) IL‐6 Concentration in MS male and female mice. (B) IL‐6 concentration in ApoE^**(−/−)**^ male and female mice. Statistical significance was determined by one‐way ANOVA, Tukey's post hoc test. The results are presented as means ± SD (*n* = 7–8). ANOVA: analysis of variance; ApoE^−/−^: apolipoprotein E; IL‐6: interleukin‐6; MS: metabolic syndrome; SD: standard deviation.
**Table S1:** Details of bioactive compounds identified in WMex, after LC‐HRMS analysis in positive ion mode.
**Table S2:** Details of bioactive compounds identified in WMex after LC‐HRMS analysis in negative ion mode.
**Table S3:** Details of bioactive compounds identified in BBex, after LC‐HRMS analysis in positive ion mode.
**Table S4:** Details of bioactive compounds identified in BBex, after LC‐HRMS analysis in negative ion mode.
**Table S5:** Evaluation of toxicity markers and hematology parameters in male mice after the 4‐week treatment. ALT: alanine aminotransferase; AST: aspartate aminotransferase. Statistical significance was determined by two‐way ANOVA, Tukey's post hoc test. The results are presented as means ± SD.
**Table S6:** Evaluation of toxicity markers and hematology parameters in female mice after the 4‐week treatment.

## Data Availability

The data that support the findings of this study are available from the corresponding author upon reasonable request.
