# Supplementary material for: Preclinical Evaluation of Berry Extracts as a Nutritional Intervention to Alleviate Myocardial Ischemia–Reperfusion Injury and Atherosclerosis Development
Source: Phytother Res. 2026 May 29;40(8):5299–315. doi: 10.1002/ptr.70391 (PMC13436189; doi:10.1002/ptr.70391)
Supplement: Supplementary file 1 [file PTR-40-5299-s001.docx]

# Supplementary Material

**Preclinical evaluation of berry-extracts as a nutritional intervention to alleviate myocardial ischemia-reperfusion injury and atherosclerosis development**

Lydia Symeonidi^a^, Panagiota-Efstathia Nikolaou^a,b^, Panagiotis Stathopoulos^c^, Andriana Christodoulou^a^, Alexandra Svouraki^c^, Panagiotis Efentakis^a^, Christina Chania^a^, Angeliki Choustoulaki^a,b^, Maria Xenaki^c^, Anna Agapaki^d^, Maria Tsoumani^a^, Nikolaos Kostomitsopoulos^e^, Ioulia Tseti^f^, Gemma Vilahur^g^, Alexios-Leandros Skaltsounis^c^, Ioanna Andreadou^a+^

*a. Laboratory of Pharmacology, Department of Pharmacy, National and Kapodistrian University of Athens, Panepistimioupolis, Zografou, Athens, Greece*

*b. Department of Clinical Therapeutics, Department of Medicine, School of Health Sciences, National and Kapodistrian University of Athens, Athens, Greece*

*c. Division of Pharmacognosy and Natural Products Chemistry, Department of Pharmacy, National and Kapodistrian University of Athens, Athens, Greece*

*d. Histochemistry Unit, Biomedical Research Foundation, Academy of Athens (BRFAA), Athens, Greece*

*e. Centre of Clinical Experimental Surgery and Translational Research, Biomedical Research Foundation of the Academy of Athens (BRFAA), Athens, Greece*

*f. Uni-Pharma S.A., Athens, Greece*

*g Research Institute Sant Pau, IIB-Sant Pau, CIBERCV, Barcelona, Spain.*

+ *With deep appreciation, we dedicate this manuscript to our esteemed colleague, Professor Ioanna Andreadou, whose contribution to this work has been invaluable. We are grateful for her friendship and the inspiration she has generously shared with us.*

**^#^Corresponding author**: Dr. Panagiotis Stathopoulos, Department of Pharmacognosy and Natural Products Chemistry, Department of Pharmacy, National and Kapodistrian University of Athens, Panepistimioupolis, Zografou, 15771, Athens, GREECE; tel: +30-210-7274886; fax: +30 210 7274827; e-mail: [stathopan@pharm.uoa.gr](javascript:void(0))

## **Supplementary methods**

## **Reagents and materials**

All reagents, HPLC-grade solvents, analytical-grade isopropanol and macroporous crosslinked polymer resin, Amberlite XAD-7, used for the experiments were purchased from Sigma Aldrich (via Life Science Chemilab SA, Athens, Greece). Similarly, the reagents that were used for the determination of total flavonoids, phenolic compounds, and anthocyanins in berry extracts. The antibodies used for Western blot analysis were purchased from Cell Signaling (via Bioline, Athens, Greece), Invitrogen (via ANTISEL, Athens, Greece) and Santa Cruz (via BioInnotech, Athens, Greece). The Western Diet was purchased from Ssniff Spezialdiäten GmbH. The PVDF membrane and ECL were purchased from Millipore (via LabSupplies, Athens, Greece).

## **Berries collection**

White Mulberries (*Morus alba*, Moraceae) were collected from the Attica region of Greece in June 2022, while Blueberries (*Vaccinium myrtillus* L., Ericaceae) originated from the Pierian Mountains, Greece, and were collected during the same harvest year. Immediately after collection the berry fruits were transferred to -80°C, where they remained for one day before being freeze-dried, using a laboratory Freeze-Dryer (Gellert Freeze Dryer Cryo -5, Langweid am Lech, Germany).

## **White Mulberry enriched extract production**

1 kg of naturally dried white mulberries was pulverized, and the resulting powder was extracted with **8 L of a solvent mixture** consisting of **H₂O/Acetic Acid** in a **ratio of 99.5/0.5 (v/v).** The extraction was carried out in an **ultrasonic reactor** for **30 minutes**. Afterward, the mixture was **centrifuged**, and the **supernatant solution** was filtered to remove suspended particles. Next, 5 kg of activated XAD7 resin were added to the aqueous extract and stirred overnight. The aim of this process was to absorb the phenolic compounds onto the surface of the resin, removing non-active components (e.g., sugars, polysaccharides, polymers). Then, after filtration, the resin was washed twice with water and transferred along with 8 L of 80° isopropanol to an ultrasonic device, where it remained for 30 minutes, to recover the XAD7 -bound phenolic compounds. The desorption process was repeated once more using an additional 5 L of 80° isopropanol. Next, the resin was filtered, and the hydroalcoholic extracts were combined. Finally, the combined alcoholic extracts concentrated in a rotary evaporator under reduced pressure, and 18.28 g of an enriched white mulberry extract (WMex) were obtained, with a **total yield of 1.82%.**

## **Blueberry enriched extract production**

**4.2 kg of fresh blueberries** were lyophilized, yielding **1.2 kg of dried fruit.** The dried fruit was then pulverized using a **laboratory blender** and extracted with **8 L of a solvent mixture** consisting of **Isopropanol/H₂O/Acetic Acid** in a **ratio of 60/39.5/0.5 (v/v/v).** The extraction was carried out in an **ultrasonic reactor** for **30 minutes**. Afterward, the mixture was **centrifuged**, and the **supernatant solution** was filtered to remove suspended particles. After removing the alcoholic solvent by evaporation, 5 L of water and 5 kg of activated XAD7 resin were added to the aqueous extract and stirred overnight. The aim of this step was to absorb the phenolic compounds onto the surface of the resin and remove non-active components (e.g., sugars, polysaccharides, polymers). Then, after filtration, the resin was washed twice with water and transferred along with 8 L of 80° isopropanol to an ultrasonic device, where it remained for 30 minutes, to recover the XAD7 -bound phenolic compounds. The desorption process was repeated once more using an additional 5 L of 80° isopropanol. Next, the resin was filtered, and the hydroalcoholic extracts were combined. Finally, the combined alcoholic extracts concentrated in a rotary evaporator under reduced pressure, and 21.16 g of an enriched blueberry extract (BBex) were obtained, with **a total yield of 1.76%**.

**Total Phenolic Content (TPC) Determination**

Total phenolic content (TPC) was measured by the colorimetric Folin–Ciocalteu assay using a 96-well microplate reader (Infinite M200, Tecan). Folin–Ciocalteu method is a photometric method that is widely used for the determination of total phenolic components in plant extracts, allows the parallel study of many samples, while at the same time it is characterized by good repeatability and low cost. Specifically, the determination of the phenolic content is based on the Folin-Ciocalteu reagent, which is a mixture of phosphotungstic acid (H_3_PW_12_O_40_) and phosphomolybdic acid (H_3_PMo_12_O_40_). This mixture interacts with the phenolic compounds resulting in the formation of a characteristic blue compound that absorbs at 765 nm. A crucial parameter of the reaction is the pH, which is adjusted by adding saturated sodium carbonate solution (Na_2_CO_3_) in the reaction solution. Gallic acid (GA) was used as a reference compound. Briefly, 25 μl of each extract (final concentration of 0.4 mg/ml) were placed in 96-well plates and then 125 μl of a mixture of deionised water and Folin–Ciocalteu reagent, in a ratio of 9/1 (v/v), was added. Exactly 3 min later, 125 μl of Na_2_CO_3_ solution (7.5% w/v) were added, and after one hour in the dark, the absorption was measured at 765 nm. Gallic acid (GA) was used to obtain a calibration curve in the range from 2.5 to 40.0 mg/L (R^2^ ≥ 0.9997). The measurement for each sample was done in triplicate, and the results were expressed as mg GA/g extract. Applying the methodology described above, the **total phenolic content** in the **WMex** and **BBex** was found **50.74** and **110.00 mg GA/g extract**, respectively.

**Total Flavonoid Content (TFC) Determination**

Total flavonoid content in the extracts was measured using the colorimetric method described by Rodrigues using a 96-well microplate reader (Infinite M200, Tecan)(Rodrigues et al., 2013). Briefly, 25 µL of each extract (final concentration of 0.4 mg/mL) were placed in 96-well plates and mixed with 100 µL of distilled water and 7.5 µL of a 5% (w/v) sodium nitrite (NaNO_2_) solution. After 5 minutes, 7.5 µL of a 10% (w/v) Aluminum Chloride (AlCl_3_) solution was added, followed by 50 µL of 1 M NaOH and 60 µL of distilled water. Subsequently, the mixture was stirred and left in the dark for 30 minutes. Absorbance was measured at 510 nm. Rutin (RUT) was used as a reference compound. The measurement for each sample was done in triplicate, and the results (mg RUT/g of extract) were expressed as the average of the three measurements. Applying the methodology described above, the **total flavonoids** in the **WMex** and **BBex** were found **49.50** and **98.92** **mg RUT/g extract**, respectively.

**Determination of Total Anthocyanin Content**

The determination of total monomeric anthocyanins was carried out according to the official method of Wrolstad using a 96-well microplate reader (Infinite M200, Tecan)(Lee et al., 2005). The pH-differential method is a rapid and simple spectrophotometric method based on the structural changes in chemical forms of anthocyanin at pH 1.0 and 4.5 (colored at pH 1.0 and colorless at pH 4.5). This method provides accurate and rapid results even in the presence of anthocyanins in the polymeric form, as they have a stable color regardless of the pH of the solution. The color change was measured spectrophotometrically, based on the difference in absorbances at the λvis-max of monomeric anthocyanins at pH 1 & 4.5.

Briefly, the pH adjustment solutions were prepared as follows:

pH 1.0 buffer (KCl, 0.025 M) preparation. 1.86 grams of KCl were weighed into a beaker, and approximately 980 milliliters of distilled water were added. The pH was then adjusted to 1.0 using HCl (37% v/v) before diluting the solution to 1 liter.

pH 4.5 buffer (CH_3_COONa, 0.4 M) preparation. 54.43 grams of CH_3_COONa were weighed into a beaker, and approximately 960 milliliters of distilled water were added. The pH was adjusted to 4.5 using HCl (37% v/v) before diluting the solution to 1 liter.

Subsequently, 1 mL of methanolic extract (1mg/mL) was mixed with 4 mL of 0.025 M hydrochloric acid (HCl) –potassium chloride (KCl) buffer (pH = 1), and then an aliquot of the resulting solution was placed in one of the wells of the 96-well plate. Similarly, 1 mL of methanolic extract (1mg/mL) was mixed with 4 mL of 0.4M sodium acetate buffer (pH = 4.5), and then an aliquot of the resulting solution was added to the adjacent well of the 96-well plate.

Absorbance measurements were performed at two wavelengths. Initially, at 520 nm (A520), which corresponds to the λvis-max of the sample's monomeric anthocyanins, and subsequently at 700 nm (A700) for correcting the turbidity of the solutions. Absorbance measurements were taken within 20-50 minutes from the sample preparation. A water/methanol mixture (80/20:v/v) was used as a blank for instrument zeroing at both wavelengths.

The content of monomeric anthocyanins (Monomeric Anthocyanin Pigment-MAP) in the extracts expressed in Cyanidin 3-glucoside was calculated using the following equation:

MAP (mg/L) = (AxMW x 1000) / (ε x l)

Where:

Α= (Α_λvis-max_-Α_700_)_pH =1_-(Α_λvis-max_-Α_700_)_pH =4.5_

MW: Molecular Weight of Cyanidin 3-glucoside (449.2 g/mol)

l: cell path length (1 cm)

ε: Molar absorptivity of Cyanidin 3-glucoside (26900 L x mol^-1^ x cm^-1^)

1000 for converting grams to milligrams.

Applying the methodology described above, the **total monomeric anthocyanin content** in the enriched **BBex** was found to be **3.05% (expressed as Cyanidin 3-glucoside)**.

**UPLC-Orbitrap MS/MS Analysis**

For the UPLC-HRMS analysis, a Waters H-Class Acquity UPLC system (Waters, Milford, MA, USA) coupled to a Velos Pro-Orbitrap Elite hybrid mass spectrometer (Thermo Scientific, USA) was utilized. Separation was performed on a Thermo Scientific™ Hypersil GOLD™ C-18 reverse phase column (100 mm x 2.1 mm, 3.0 µm), with a stable temperature of 40°C. The flow rate was set at 400 μL/min and the solvent system was (A) water 0.1% Formic Acid and (B) acetonitrile. The gradient elution program started with 5% of B which held for 1 minute. In the next 14 minutes B reached 100% and held for 2 minutes. Finally, the system returned to the initial conditions for system equilibration. The injection volume was 10 μL and the autosampler temperature was set at 10°C.

Mass spectra were obtained in positive and negative ion mode with a heated ion electrospray (HESI) source. The HESI conditions were as follows: capillary and source heater temperature were set at 350 °C; source voltage for positive ion mode at 3.6 kV and for negative at 2.7kV. Sheath and auxiliary gas were adjusted to 45 and 15 arbitrary units, respectively.

The HRMS data were acquired in full scan mode in the range of 113-1000 m/z, with resolving power 60,000 at 500 m/z and scan rate 1 microscan per second. HRMS/MS experiments were obtained in data-depending method with collision energy 35.0% (q = 0.25). The raw data were acquired and processed with Thermo Xcalibur Version 2.2. Spectrometric features were used for identification of the constituents, such as accurate m/z, proposed elemental composition (EC), observed isotopic patterns and ring double bond equivalent (RDBeq) values. The HRMS/MS experiments, in combination with existing in-house and online databases such as the METLIN Metabolomics Database (https://metlin.scripps.edu/) and the ChemSpider database (http://www.chemspider.com/), significantly contributed to the determination of the structures of the components of each extract. The data were recorded at the end of the process and compared with literature data. The results of LC-HRMS analysis showed that the **WMex** was rich in **acids** (quinic acid, malic acid, citric acid), **phenolic acids** (chlorogenic acid, 3-p-coumaroylquinic acid), **pyrrolidine alkaloids** (isomers of morusimic acids B, C, E), and **flavonoids** (rutin, quercetin, kaempferol, isoquercitrin, quercetin, as aglycones or with their corresponding glycosides). Meanwhile, in the BBex, the main detected secondary metabolites were **anthocyanins** (delphinidin-3-O-galactoside, delphinidin-3-O-arabinoside, petunidin-3-O-galactoside, cyanidin-3-O-arabinoside, petunidin-3-O-arabinoside, malvidin-3-O-galactoside, malvidin-3-O-glucoside, malvidin-acetyl-hexoside), **acids** (quinic acid, malic acid, citric acid), **phenolic acids** (gallic acid, caffeic acid hexoside, caffeic acid, chlorogenic acid, ferulic acid hexoside, ferulic acid, 3-feruloylquinic acid), and **flavonoids** (rutin, isoquercitrin, myricetin, quercetin, kaempferol, quercetin, quercitrin, gossypetin, isorhamnetin, syringetin, as aglycones or with their corresponding glycosides). (Supplementary Figures 1-4 and Supplementary Tables 1-4)

**HPLC-DAD Analysis**

The qualitative determination of polar-phenolic compounds in both berry extracts were performed by applying an in-house analytical method. Specifically, the separation of the components of the extracts was achieved on a reversed-phase Discovery HS C18 column (250 × 4.6 mm, 5 µm) using a mobile phase consisting of 0.3% aqueous orthophosphoric acid (A) and ACN (acetonitrile) (B), at a flow rate of 1.0 mL/min and ambient temperature. The applied gradient elution was as follows: 0 min, 90% A and 10% B; 20 min, 83% A and 17% B; 40 min, 83% A and 17% B; 43 min, 90% A and 10% B; 45 min, 90% A and 10% B. The injection volume was held constant at 20 µL, and chromatograms were monitored at 280 nm and 520 nm. As we can see in the HPLC-DAD chromatograms (Supplementary Figures 5 and 6), recorded at 525 nm (characteristic absorption wavelength of anthocyanins), the **BBex was rich in anthocyanins**, in contrast to the **WMex**, where anthocyanin levels were below the detection limit of the HPLC-DAD method.

## **Cryosections**

Freshly isolated aorta samples were immersed in OCT embedding medium tissue-tek by immediate freezing in liquid nitrogen. Using a cryostat (Leica microsystems GmbH, Wetzlar, Germany), 8 μm thick frozen aorta cross sections were collected onto labeled Poly-L-lysine-coated glass slides (Fisherbrand™ Superfrost™ Plus). The glass slides were left to air dry, then fixed with 4% paraformaldehyde for 5 minutes and washed with 1X PBS for 5 minutes. Following this, they were washed with 60% isopropanol for 5 minutes and stained with Oil Red O for 20 minutes. Then, they were washed again with isopropanol 3 times/2sec and mounting medium (Diamount mounting medium, DiaPath®) was placed on the aortic sections to quantify the atherosclerotic plaque area using ImageJ software, thus, to evaluate the atherosclerotic lesion as the percentage of the total aortic area.

## **Murine model of myocardial IR**

Mice were anesthetized with a combination of ketamine (100 mg/kg), xylazine (20 mg/kg) and atropine (0.6 mg/kg) and intraperitoneal administration was performed and remained under general anesthesia during the IRI procedures. The proper plane of anesthesia was reached when pedal reflex was lost. Afterwards, body temperature was sustained in all animals at 37℃ using a heating pad and a tracheal tube was placed via tracheotomy to ensure artificial respiration, using a MiniVent ventilator for mice (Model 845, Harvard Apparatus), set at 150 strokes/min with a tidal volume of 200 μL. A left sided thoracotomy was performed revealing the heart, the pericardium was removed and LAD was ligated 3-4 mm distal to the origin of the artery under the left atrium using a 6-0 silk suture (medipac manufacturing). Ischemia was then induced for 30 min by tightening the suture using a small piece of polyethylene tube to ensure coronary circulation blockage. After 30 min of ischemia, the ligation was released for 2 hours to allow reperfusion. Animals of the 1^st^ and 2^nd^ experimental protocol remained under general until the end of the reperfusion and were subsequently euthanized by cervical dislocation and the heart was gently excised (Bøtker et al., 2018; Nikolaou et al., 2022).

## **Murine heart staining for infarct size measurement**

Infarct size was evaluated by double staining of the heart with Evans Blue dye (MP Biochemicals, LLC, #151108) and 2,3,5-Triphenyltetrazolium chloride (TTC) solution. In more detail, the aorta was cannulated and perfused with Krebs buffer (NaCl 118.5 mM, NaHCO_3_ 25.0 mM, KCl 47.0 mM, KH_2_PO_4_ 12.0 mM, MgSO_4_ 12.0 mM, Glucose 11.1 mM, CaCl_2_ 2.4 mM) until excess blood was removed from the myocardium. The suture was then re-tightened and 2% Evans Blue dye in Krebs buffer was slowly infused into the aorta, to separate the ischemic part of the myocardium from the non-occluded, normally perfused area. The hearts were sliced in 1 mm thick sections and incubated in 1% TTC in PBS solution (pH 7.4) at 37℃ for 10 min, thus, the viable tissue was stained red whereas necrotic area was determined as white. Pictures of the heart slices were obtained (Olympus EP50 digital camera on Olympus SZ61 stereoscope) and the overall size of each slice (A) as well as the area at risk and infarct size were determined using ImageJ software, therefore, the percentages of area at risk and I/R% were evaluated (Nikolaou et al., 2022).

## **Blood glucose levels**

Fasting blood glucose levels were determined after a 16-hour overnight fasting by collecting a blood drop from the tail vein using a blood glucose meter Accu-Chek Instant (Roche Diabetes Care GmbH).

## **Lipid profile and toxicity biomarkers in plasma**

Blood was collected from the submandibular vein and heparin (1:6 in normal saline) was used as an anticoagulant (heparin: blood, 1:10 v/v). The blood samples were then centrifuged at 5000 x g for 15 minutes and plasma was collected. Total Cholesterol (#000218), Triglycerides (#000243), High-Density Lipoprotein (#000238), Aspartic Aminotransferase (AST) (#1695), Alanine Aminotransferase (ALT) (#001696), Creatinine (#000216) and Urea (#001526) were measured using commercially available assay kits purchased by Biotechnological Applications Ltd and all measurements were performed per manufacturers’ instructions. Low-density lipoprotein (LDL) was calculated using a mathematical formula. Blood was analyzed by an Automated Hematology Analyzer (Sysmex pocH-100iV Diff) to determine the number of cells (White and Red Blood Cells and Platelets), Hematocrit and Hemoglobin.

## **Western Blot analysis of aorta and ischemic myocardial tissue**

Western blot analysis was performed as previously described (Nikolaou et al., 2022). Myocardial tissue samples were harvested at the 10^th^ min of reperfusion, the ischemic part was collected, and protein content was isolated using RIPA Lysis Buffer (1% Triton x 100, 20 mM Tris pH 7.4-7.6, 150 mM NaCl, 50 mM NaF, 1mM EDTA, 1mM ethylene glycol tetraacetic acid, 1 mM glycerolphosphatase, 1% sodium dodecyl sulfate (SDS), 100 mM phenylmethanesulfonyl fluoride and 0.1% protease phosphatase inhibitor cocktail). The samples were then centrifuged at 11,000 x g for 15 min at 4℃ and protein content was quantified using Lowry Protein assay. An equal amount of protein was used to prepare Western blot samples by using Dave’s buffer (4% SDS, 10% 2-mercaptoethanol, 20% glycerol, 0.004% bromophenol blue and 0.125M Tris-HCl) and were then boiled at 100℃ for 10 min. The samples were separated by 10%, 12% or 15% SDS-polyacrylamide gel electrophoresis using a colored protein ladder (BlueStar Plus Prestained Protein Marker, Nippon Genetics®), transferred onto a polyvinylidene difluoride (PVDF) membrane (P 0.45 μm Polyvinylidene difluoride membrane*,* Cytiva Amersham™), immunoblotted and blocked with 5% nonfat dry milk or 5% Bovine Serum Albumin (BSA). Membranes were then incubated overnight at 4℃ with the following primary antibodies: endothelial-nitric oxide synthase (eNOS) (Rabbit mAb #32027, dilution 1:1000, Lot:5), phospho-nuclear factor kappaB Ser536 (p-NF-κB) (Rabbit mAb #3033, dilution 1:1000, Lot:19), nuclear-factor kappaB (t-NF-κB) (Mouse mAb#6956, dilution 1:1000, Lot:10), phospho-STAT3 Tyr705 (p-STAT3) (Rabbit mAb#9145, dilution 1:1000, Lot:43), STAT3 (t-STAT3) (Mouse mAb#9139, dilution: 1:1000, Lot:16) , (phospho-Protein Kinase B Ser473 (p-Akt) (Rabbit mAb #9271, dilution 1:1000, Lot:15), Protein Kinase B (t-Akt) (Rabbit mAb #9272, dilution 1:1000, Lot:30), Glycogen Synthase Kinase 3 beta (GKS-3β) (Rabbit mAb #9315, dilution 1:1000, Lot:14), phospho-p44/42 MAPK (p-Erk1/2) T202/Y204 (Mouse mAb #9106, dilution 1:1000, Lot:38), p44/42 MAPK (Erk1/2) (Mouse mAb #9107, dilution 1:1000, Lot:10), Nucleotide-binding oligomerization domain -like receptor 3 (NLRP3) (Rabbit mAb #15101, dilution 1:500, Lot:8), Bax (Rabbit mAb #14796, dilution 1:1000, Lot:2), B-cell lymphoma-extra large (Bcl-xL) (Rabbit mAb #2764, dilution 1:1000, Lot:11), phospho-Vasodilator-Stimulated Phosphoprotein S239 (p-VASP) (Rabbit mAb#3114, dilution 1:1000, Lot:7), Vasodilator-Stimulated Phosphoprotein (VASP) (Rabbit mAb#3132, dilution 1:1000, Lot:5), Glyceraldehyde-3-Phosphate Dehydrogenase (GAPDH) (Rabbit mAb #5174, dilution 1:2500, Lot:8), α-Actinin (Rabbit mAb #6487, dilution 1:2000, Lot:4), β-Tubulin (Rabbit mAb #2146, dilution 1:2000, Lot:10) purchased from Cell Signaling Technology, Gasdermin (Mouse mAb#sc-376318, dilution 1:1000, Lot:K1521), Caspase-1 (Mouse mAb#sc-56036, dilution 1:1000, Lot:L1218), phospho-Glycogen Synthase Kinase beta Ser9 (GSK-3β) (Mouse mAb #sc-373800, dilution 1:1000, Lot:J1017) purchased from Santa Cruz Biotechnology, purified Anti-eNOS Ser1177 (p-eNOS) (Mouse mAB#612393, dilution 1:1000, Lot:0328546) purchased from BD Biosciences and Intercellular Adhesion Molecule-1 (ICAM-1) (Mouse mAb#1A29, dilution 1:1000,) purchased from Invitrogen. Membranes were then incubated for 1-2 hrs at room temperature with the following secondary antibodies: Anti-rabbit IgG, HRP-linked antibody #7074, dilution 1:2000, Lot:33 or Anti-mouse IgG, HRP-linked Antibody #7076, dilution 1:2000, Lot:36 purchased from Cell Signaling Technology. Protein detection was performed by chemiluminescence using ImageQuant LAS 500 after exposure to the HRP substrate, ECL (Immobilon Forte Western HRP substrate, Millipore). Relative densitometry was determined using GelPro Analyzer 4.0 software and all values were normalized to the respective control-treated group. GAPDH, α-Actinin and β-tubulin were used as loading control proteins (Nikolaou et al., 2021).

**Serum Biomarkers in vivo**

Serum biomarkers were determined using commercial kits. Interleukin-6 (IL-6) (Murine IL-6 ELISA kit, CAT Number: EA-101689) was determined using commercially available ELISA kits purchased from OriGene Technologies, Inc. (via LabSupplies Scientific, Athens, Greece) according to manufacturer’s instructions. Estradiol (E2) (QuicKey Pro Mouse E2 (Estradiol) ELISA kit, CAT Number: E-OSEL-M0008) was determined using commercially available ELISA kits purchased from Elabscience (via BioInnotech, Athens, Greece) according to manufacturer’s instructions.

**Circulating nitrite measurement**

The concentration of nitrite in blood plasma was determined using Griess reaction with a commercially available kit (Cayman's Nitrate/Nitrite Colorimetric Assay Kit 780001) as we have previously described (Bibli et al., 2019). Each plasma sample was ultrafiltered through a 30kDa molecular weight cutoff filter purchased from Millipore USA (Amicon Ultra 0.5 mL, 30 kDa via LabSupplies, Athens, Greece, UFC503096). The filters were pre-rinsed with Ultrapure water prior to ultrafiltration of the plasma. Then, 500 μL of plasma was centrifuged for 30 minutes at 14.000 x g at 4℃. For the determination of nitrites, 40 μL of the filtrate was used, the Griess reagents were added, and the absorbance of each well was measured at 540 nm using the reader Infinite 200 PRO series (Tecan). The concentration of nitrite was determined with a nitrite standard curve while the concentration of nitrate was determined with a nitrate standard curve using Graph Pad prism version 9.0.0 (Graph Pad Software, Inc.) according to the manufacturer’s instructions.

**Supplementary figures**

**Supplementary Fig. 1ː** LC-HRMS chromatogram, recorded in positive ion mode, of the WMex. The identified bioactive compounds are highlighted.

**Supplementary Fig. 2ː** LC-HRMS chromatogram, recorded in negative mode, of the WMex. The identified bioactive compounds are highlighted.

**Supplementary Fig. 3ː** LC-HRMS chromatogram recorded, in positive ion mode, of the BBex. The identified bioactive compounds are highlighted.

**Supplementary Fig. 4ː** LC-HRMS chromatogram, recorded in negative ion mode, of the BBex. The identified bioactive compounds are highlighted.

**Supplementary Fig. 5ː** HPLC-DAD chromatogram, recorded at 525 nm of the BBex.

**Supplementary Fig. 6ː** HPLC-DAD chromatogram, recorded at 525 nm of the WMex.

**Supplementary Fig. 7.** **Assessment of** **MS induction in C57Bl6 mice fed on Western diet (WD) with a cluster of metabolic factors such as body weight, blood glucose levels, total cholesterol and triglycerides. A.** Effect of WD on body weight in male and female mice. **B.** Effect of WD on blood glucose levels in both sexes and **C.** Effect of WD on total cholesterol and triglyceride levels in both sexes. Statistical significance was determined by one-way ANOVA, Tukey’s post-hoc test. **p<0.05, **p<0.01, ***p<0.001, ****p<0.0001*. The results are presented as means ± SD (n=8). MS: Metabolic Syndrome; ANOVA: Analysis of Variance; SD: Standard Deviation.

**Supplementary Figure. 8.** **Effect of 8-week supplementation with berry extracts on body weight and blood glucose levels in Western diet (WD) fed C57Bl6 mice.** **A.** Body weight in male mice. **B.** Blood glucose levels in male mice. **C.** Lipid profile in male mice. **D.** Body weight in female mice. **E.** Blood glucose levels in female mice and **F.** Lipid profile in female mice. Statistical significance was determined by one-way ANOVA, Tukey’s post-hoc test for Fig. B,C,D,E,F and Kruskal-Wallis, Dunn’s post-hoc test, for Fig. A. The results are presented as means ± SD (n=6-8). ANOVA: Analysis of Variance; SD: Standard Deviation.

**Supplementary Fig. 9.** **Assessment of MS induction with a cluster of metabolic factors such as body weight, blood glucose, total cholesterol and triglycerides in Western diet (WD) fed ApoE^(-/-)^ mice. A.** Effect of WD on body weight in male and female mice. **B.** Effect of WD on blood glucose levels in both sexes and **C.** Effect of WD on total cholesterol and triglyceride levels in both sexes. Statistical significance was determined by one-way ANOVA, Tukey’s post-hoc test. **p<0.05, **p<0.01, ***p<0.001, ****p<0.0001*. The results are presented as means ± SD (n=8). MS: Metabolic Syndrome; ApoE**-/-**: Apolipoprotein E; ANOVA: Analysis of Variance; SD: Standard Deviation.

**Supplementary Fig. 10.** **Effect of 4-week supplementation with berry extracts on body weight and lipid levels in Western diet (WD) fed ApoE^(-/-)^ mice.** **A.** Body weight in both sexes, **B.** HDL levels in both sexes and **C.** LDL levels in both sexes. Statistical significance was determined by two-way ANOVA, Tukey’s post-hoc test. The results are presented as means ± SD (n=8). ApoE**-/-**: Apolipoprotein E; HDL: High-Density Lipoprotein; LDL: Low-Density Lipoprotein; ANOVA: Analysis of Variance; SD: Standard Deviation.

**Supplementary Fig. 11. Effect of IL-6 inflammatory biomarker in both mice with MS and ApoE^(-/-)^. A.** IL-6 Concentration in MS male and female mice. **B.** IL-6 Concentration in ApoE**^(-/-)^** male and female mice. Statistical significance was determined by one-way ANOVA, Tukey’s post-hoc test. The results are presented as means ± SD (n=7-8). IL-6: Interleukin-6; MS: Metabolic Syndrome; ApoE**-/-**: Apolipoprotein E; ANOVA: Analysis of Variance; SD: Standard Deviation.

**Supplementary Tables**

**Supplementary Table 1ː** Details of bioactive compounds identified in WMex, after LC-HRMS analysis in positive ion mode.

**Supplementary Table 2ː** Details of bioactive compounds identified in WMex after LC-HRMS analysis in negative ion mode

**Supplementary Table 3ː** Details of bioactive compounds identified in BBex, after LC-HRMS analysis in positive ion mode.

**Supplementary Table 4ː** Details of bioactive compounds identified in BBex, after LC-HRMS analysis in negative ion mode

**Supplementary Table 5.** Evaluation of toxicity markers and hematology parameters in male mice after the 4-week treatment. AST: Aspartate Aminotransferase; ALT: Alanine Aminotransferase. Statistical significance was determined by two-way ANOVA, Tukey’s post-hoc test. The results are presented as means ± SD.

|  | Control  (5% DMSO) | WMex  500 mg/kg | p value  vs Control (5% DMSO) | ΒΒex  500 mg/kg | p value  vs Control (5% DMSO) |
| --- | --- | --- | --- | --- | --- |
| Toxicity markers in serum |  |  |  |  |  |
| AST (U/L) | 47.07 ± 3.17 | 45.67 ± 0.37 | 0.9996 | 43.44 ± 0.07 | 0.9947 |
| ALT (U/L) | 17.18 ± 1.18 | 18.39 ± 1.43 | 0.9886 | 18.20 ± 1.65 | 0.9605 |
| Creatinine (mg/dL) | 0.344 ± 0.044 | 0.356 ± 0.032 | 0.4886 | 0.355 ± 0.011 | 0.8772 |
| Urea (mg/dL) | 50.71 ± 2.80 | 52.81 ± 2.92 | 0.9126 | 52.01 ± 2.47 | 0.3791 |
| Fasting Glucose (mg/dL) | 67.43 ± 0.60 | 66.93 ± 5.55 | 0.5425 | 69.86 ± 4.84 | 0.9974 |
| Body weight (g) | 25.63 ± 0.88 | 27.56 ± 0.10 | 0.7497 | 27.07 ± 0.30 | 0.8751 |
| Hematological parameters |  |  |  |  |  |
| White Blood Cells (x10^3^/μL) | 10.12 ± 1.50 | 9.31 ± 1.88 | 0.4347 | 8.98 ± 2.16 | 0.1650 |
| Red Blood Cells (x10^6^/μL) | 10.26 ± 0.11 | 10.37 ± 0.21 | 0.9966 | 10.37 ± 0.07 | 0.8300 |
| Hemoglobin (g/dL) | 12.56 ± 0.00 | 12.20 ± 0.56 | 0.2783 | 12.41 ± 0.04 | 0.9341 |
| Hematocrit (%) | 59.01 ± 2.92 | 58.76 ± 1.85 | 0.9788 | 59.03 ± 0.20 | 0.8480 |
| Platelets (x10^3^/μL) | 1099 ± 130.4 | 1309 ± 114.9 | 0.1337 | 1260 ± 119.3 | 0.2981 |

**Supplementary Table 6.** Evaluation of toxicity markers and hematology parameters in female mice after the 4-week treatment.

|  | Control  (5% DMSO) | WMEX  500 mg/kg | p value  vs Control (5% DMSO) | BLB_16_XAD7  500 mg/kg | p value  vs Control (5% DMSO) |
| --- | --- | --- | --- | --- | --- |
| Toxicity markers in serum |  |  |  |  |  |
| AST (U/L) | 48.65 ± 1.92 | 47.43 ± 3.76 | 0.9067 | 49.72 ± 4.64 | 0.6833 |
| ALT (U/L) | 42.14 ± 1.21 | 39.18 ± 4.63 | 0.9584 | 43.84 ± 1.65 | 0.1621 |
| Creatinine (mg/dL) | 0.502 ± 0.039 | 0.470 ± 0.151 | 0.1252 | 0.471 ± 0.024 | 0.2294 |
| Urea (mg/dL) | 31.91 ± 2.22 | 31.67 ± 1.88 | 0.6605 | 38.02 ± 0.40 | 0.4946 |
| Fasting Glucose (mg/dL) | 65.63 ± 2.29 | 65.56 ± 0.26 | 0.9448 | 64.75 ± 0.53 | 0.9994 |
| Body weight (g) | 21.49 ± 0.33 | 21.11 ± 0.15 | 0.6427 | 21.89 ± 0.22 | 0.8285 |
| Hematological parameters |  |  |  |  |  |
| White Blood Cells (x10^3^/μL) | 8.29 ± 1.79 | 7.51 ± 1.75 | 0.5471 | 7.81 ± 0.77 | 0.2750 |
| Red Blood Cells (x10^6^/μL) | 10.27 ± 0.54 | 9.72 ± 0.73 | 0.3926 | 10.10 ± 0.35 | 0.9975 |
| Hemoglobin (g/dL) | 12.99 ± 0.37 | 12.63 ± 0.33 | 0.8700 | 12.84 ± 0.11 | 0.9983 |
| Hematocrit (%) | 55.78 ± 2.92 | 53.86 ± 1.85 | 0.6091 | 55.29 ± 0.20 | 0.9682 |
| Platelets (x10^3^/μL) | 1062 ± 113.6 | 1002 ± 217.6 | 0.9665 | 1104 ± 238.9 | 0.0553 |

# **References**

Bibli, S.-I., Papapetropoulos, A., Iliodromitis, E.K., Daiber, A., Randriamboavonjy, V., Steven, S., Brouckaert, P., Chatzianastasiou, A., Kypreos, K.E., Hausenloy, D.J., Fleming, I., Andreadou, I., 2019. Nitroglycerine limits infarct size through S-nitrosation of cyclophilin D: a novel mechanism for an old drug. Cardiovascular Research 115, 625–636. https://doi.org/10.1093/cvr/cvy222

Bøtker, H.E., Hausenloy, D., Andreadou, I., Antonucci, S., Boengler, K., Davidson, S.M., Deshwal, S., Devaux, Y., Di Lisa, F., Di Sante, M., Efentakis, P., Femminò, S., García-Dorado, D., Giricz, Z., Ibanez, B., Iliodromitis, E., Kaludercic, N., Kleinbongard, P., Neuhäuser, M., Ovize, M., Pagliaro, P., Rahbek-Schmidt, M., Ruiz-Meana, M., Schlüter, K.-D., Schulz, R., Skyschally, A., Wilder, C., Yellon, D.M., Ferdinandy, P., Heusch, G., 2018. Practical guidelines for rigor and reproducibility in preclinical and clinical studies on cardioprotection. Basic Research in Cardiology 113, 39. https://doi.org/10.1007/s00395-018-0696-8

Lee, J., Durst, R.W., Wrolstad, R.E., Collaborators:, Eisele, T., Giusti, M.M., Hach�, J., Hofsommer, H., Koswig, S., Krueger, D.A., Kupina;, S., Martin, S.K., Martinsen, B.K., Miller, T.C., Paquette, F., Ryabkova, A., Skrede, G., Trenn, U., Wightman, J.D., 2005. Determination of Total Monomeric Anthocyanin Pigment Content of Fruit Juices, Beverages, Natural Colorants, and Wines by the pH Differential Method: Collaborative Study. Journal of AOAC INTERNATIONAL 88, 1269–1278. https://doi.org/10.1093/jaoac/88.5.1269

Nikolaou, P.E., Efentakis, P., Abu Qourah, F., Femminò, S., Makridakis, M., Kanaki, Z., Varela, A., Tsoumani, M., Davos, C.H., Dimitriou, C.A., Tasouli, A., Dimitriadis, G., Kostomitsopoulos, N., Zuurbier, C.J., Vlahou, A., Klinakis, A., Brizzi, M.F., Iliodromitis, E.K., Andreadou, I., 2021. Chronic Empagliflozin Treatment Reduces Myocardial Infarct Size in Nondiabetic Mice Through STAT-3-Mediated Protection on Microvascular Endothelial Cells and Reduction of Oxidative Stress. Antioxidants & redox signaling 34, 551–571.

Nikolaou, P.E., Mylonas, N., Makridakis, M., Makrecka-Kuka, M., Iliou, A., Zerikiotis, S., Efentakis, P., Kampoukos, S., Kostomitsopoulos, N., Vilskersts, R., Ikonomidis, I., Lambadiari, V., Zuurbier, C.J., Latosinska, A., Vlahou, A., Dimitriadis, G., Iliodromitis, E.K., Andreadou, I., 2022. Cardioprotection by selective SGLT-2 inhibitors in a non-diabetic mouse model of myocardial ischemia/reperfusion injury: a class or a drug effect? Basic Research in Cardiology 117, 27. https://doi.org/10.1007/s00395-022-00934-7

Rodrigues, F., Palmeira-de-Oliveira, A., Das Neves, J., Sarmento, B., Amaral, M.H., Oliveira, M.B., 2013. Medicago spp. extracts as promising ingredients for skin care products. Industrial Crops and Products 49, 634–644. https://doi.org/10.1016/j.indcrop.2013.06.015
